# Randomized clinical trial on the efficacy of intranasal or oral ketamine-midazolam combinations compared to oral midazolam for outpatient pediatric sedation

**DOI:** 10.1371/journal.pone.0213074

**Published:** 2019-03-11

**Authors:** Joji Sado-Filho, Karolline Alves Viana, Patrícia Corrêa-Faria, Luciane Rezende Costa, Paulo Sucasas Costa

**Affiliations:** 1 Health Sciences Graduate Program, Faculdade de Medicina, Universidade Federal de Goiás, Goiânia, Goiás, Brazil; 2 Dentistry Graduate Program, Faculdade de Odontologia, Universidade Federal de Goiás, Goiânia, Goiás, Brazil; 3 Department of Oral Health, Faculdade de Odontologia, Universidade Federal de Goiás, Goiânia, Goiás, Brazil; 4 Department of Pediatrics, Faculdade de Medicina, Universidade Federal de Goiás, Goiânia, Goiás, Brazil; University of Bern, SWITZERLAND

## Abstract

**Purpose:**

The optimal sedative regime that provides the greatest comfort and the lowest risk for procedural sedation in young children remains to be determined. The aim of this randomized, blinded, controlled, parallel-design trial was to evaluate the efficacy of intranasal ketamine and midazolam as the main component of the behavioral guidance approach for preschoolers during dental treatment.

**Materials and methods:**

Children under seven years of age, with caries and non-cooperative behavior, were randomized into three groups: (KMIN) intranasal ketamine and midazolam; (KMO) oral ketamine and midazolam; or (MO) oral midazolam. The dental sedation appointments were videotaped, and the videos were analyzed using the Ohio State University Behavioral Rating Scale (OSUBRS) to determine the success of the sedation in each group. Intra- and postoperative adverse events were recorded. Data analysis involved descriptive statistics and non-parametric tests (*P* < 0.05, IBM SPSS).

**Results:**

Participants were 84 children (28 per group; 43 boys), with a mean age of 3.1 years (SD 1.2). Children’s baseline and the dental sedation session characteristics were balanced among groups. The success of the treatment as assessed by the dichotomous variable ‘quiet behavior for at least 60% of the session length’ was: KMIN 50.0% (n = 14; OR 2.10, 95% CI 0.71 to 6.30), KMO 46.4% (n = 13; OR 1.80, 95% CI 0.62 to 5.40), MO 32.1% (n = 9) (P = 0.360). Adverse events were minor, occurred in 37 of 84 children (44.0%), and did not differ among groups (P = 0.462).

**Conclusion:**

All three regimens provided moderate dental sedation with minor adverse events, with marked variability in the behavior of children during dental treatment. The potential benefit of the ketamine–midazolam combination should be further investigated in studies with larger samples.

**Trial registration:**

ClinicalTrials.gov, identifier: NCT02447289. Registered on 11 May 2015, named “Midazolam and Ketamine Effect Administered Through the Nose for Sedation of Children for Dental Treatment (NASO).”

## Introduction

Procedural sedation and analgesia outside of the operating room is still a challenge for many health disciplines that focus on children. One of these challenges is related to the effectiveness of sedatives [1]. In the last five years, systematic reviews have reported that “new” and “old” sedatives such as midazolam, nitrous oxide, ketamine and propofol [2], dexmedetomidine [3], and chloral hydrate [4] lack high quality evidence on efficacy and safety for administration in children in diverse settings. In this respect, the dental setting allows wide perspectives on sedative agents, as this setting combines pain, anxiety, lasting procedures, and urgent care. Anxious children suffering the negative impacts of dental caries would experience harm if they remain in queues for general anesthesia without any immediate relief [5].

Diverse sedative drugs, dosages and mode of administration have been studied in pediatric dentistry, in order to identify the safest and most effective option [6]. Midazolam is the drug most commonly used in this context, whereas other drugs such as ketamine have also demonstrated efficacy in behavioral control [7,8]. Moreover, it has been shown that administration of both ketamine and midazolam could be a good choice for pediatric dental sedation, including for very young children [9,10].

Given the need to optimize the sedative technique, different routes of drug delivery have been investigated; the intranasal route appears to be an effective option for procedural sedation [11]. Although there are some published clinical trials evaluating intranasal sedation for dental treatment of children, to the best of our knowledge, among these, only one study compared the efficacy of midazolam and ketamine as given by oral or intranasal route [12]. However, that study had a crossover nature, which can compromise the interpretation of the results in this type of study [6].

Thus, considering the lack of well-designed trials evaluating the efficacy of intranasal midazolam/ketamine in procedural sedation, the aim of this randomized, blinded, controlled, parallel-design trial was to evaluate the efficacy of intranasal sedation with ketamine and midazolam for the behavioral management of preschoolers undergoing dental treatment, as compared with oral sedation using the same combination of agents, or midazolam alone, which is considered gold standard sedative [13]. We hypothesized that intranasal sedation with midazolam and ketamine would improve children’s behavior, with minimal adverse events, compared to oral sedation.

## Materials and methods

This randomized, controlled and triple-blind clinical trial, with three parallel groups (allocation ratio 1:1:1), was approved by the Research Ethics Committee of the Universidade Federal de Goiás (UFG), Brazil (protocol CAAE 36411214.1.0000.5083). The rights of the participants were protected, and the parents/guardians signed a statement of informed consent prior to participation in this study. In this study, we report on the main outcome findings of the investigation registered in the Clinical Trials Database prior to the start of the trial and any patient enrollment undertaken (ClinicalTrials.gov NCT02447289). The full protocol of this clinical trial has been published ([14]; S1 and S2 Protocols) and complies with CONSORT statement (S1 Appendix). The full protocol included other variables that are going to be analyzed and reported in future publications: children’s pain and acceptance of the sedative administration; children’s memory of intraoperative procedures; children’s, parents’ and dentists’ stress and perceptions of sedation; economic analysis of the sedative regimes [14]. There were no changes to methods after trial commencement. The participant recruitment started on May 21, 2015 and the study was completed on October 18, 2016.

### Participants and study setting

This study was conducted in children under seven years of age. Participants had ASA physical status I or II and little risk of airway obstruction [15], no medical history of neurological or cognitive alterations, and at least two decayed teeth without pulp involvement requiring dental restoration. Recruited children had their uncooperative behavior confirmed during dental exam. If children showed positive behavior (acceptance of dental treatment) in dental exam session, they were scheduled to a dental restorative appointment without sedation. Then, if they remained behaving well, they were excluded from the study.

This study took place in an university outpatient dental sedation clinic, where a multiprofessional team provides dental sedation for people with oral problems and difficulties facing dental treatment due to anxiety or immature/deficient cognitive abilities. Trained anesthesiologists, pediatricians, psychologists, pediatric and general practice dentists, graduate and undergraduate students all follow acknowledged guidelines to provide this service [16].

### Interventions

Children were randomly assigned to one of the three groups: KMIN (experimental): combination of intranasal ketamine (4.0 mg/kg, maximum 100.0 mg) and midazolam (0.2 mg/kg, maximum 5.0 mg) [17–19]; KMO (control for the administration route): combination of ketamine (4.0 mg/kg, maximum 100.0 mg) and midazolam (0.5 mg/kg, maximum 5.0 mg) [17–20] by oral route; and MO (control): oral midazolam (1.0 mg/kg, maximum 20.0 mg) [9]. The sedative formulations were: ketamine injectable solution in a concentration of 50.0 mg/mL (Ketamin S, Cristalia, São Paulo, Brazil); midazolam injectable solution in a concentration of 5.0 mg/mL (Dormire injectable solution, Cristalia, São Paulo, Brazil), and midazolam oral solution in a concentration of 2.0 mg/mL (Dormire oral solution, Cristalia, São Paulo, Brazil).

One anesthesiologist carried out the sedative administration following a standardized sequence and time intervals between drugs (S1 Fig). First, the anesthesiologist administered the oral syrups (midazolam/ketamine, midazolam, or placebo), and then the sedative or placebo (saline) via intranasal route. For the KMIN group, the administration started with ketamine dispensed in an insulin syringe and an atomizer (LMA MAD Nasal, Teleflex, Fort Worth, TX, USA) with a maximum dose of 0.5 mL per hour, followed by midazolam after three minutes.

Seven minutes after the administration of the last sedative, the parent and child were taken to the dental office to start the dental treatment. One parent remained in the dental chair with the child's legs supported on their lap. A trained observer monitored the child throughout the appointment using a pulse oximeter (Datex-Ohmeda, Helsinki, Finland) and visual inspection. A pediatric dentist and one assistant performed one tooth restoration under local anesthesia and rubber dam isolation. Pediatric dentists used non-pharmacological techniques such as distraction, as demanded by the children. Soon after the end of the procedure, the parent/child dyad was taken to the sedation recovery room and remained there being continuously monitored by an observer until they met the discharge criteria [16].

### Outcomes

The outcomes for the present study were: primary–children’s behavior during dental sedation; secondary–occurrence of intra- and postoperative adverse events.

Child’s behavior was evaluated according to the Ohio State University Behavioral Rating Scale (OSUBRS) [21]. This scale possess four categories, as following: (1) quiet behavior, (2) crying without movement, (3) resistant movement without crying, and (4) struggling.

Four blinded examiners were trained and calibrated to evaluate the child's behavior via digital records using Observer XT software (Noldus, The Netherlands). For training, they watched seven videos from different children not included in this study and discussed children’s behavior in depth with the principal investigator. For calibration, they watched five different videos. The intra-class coefficients (ICC) for the inter-examiner agreement were: OSUBRS 1 ICC = 0.97; OSUBRS 2 ICC = 0.90; OSUBRS 3 ICC = 0.80; OSUBRS 4 ICC = 0.95. For intra-examiner agreement, eleven videos were re-evaluated by the same examiner, with an interval of about two weeks (ICC ≥ 0.994 for all examiners).

One of four previously trained and calibrated observers independently watched the videos taken during the dental treatment and assessed each child’s behavior using Observer XT software (Noldus, The Netherlands). The observer continuously registered one of the four OSUBRS scores during the whole sedation session, which generated a continuous variable for each case, named the percentage of each OSUBRS score observed in a given dental sedation session.

Adverse events were “unexpected and undesirable responses to sedatives that threaten or cause patient injury or discomfort” [10] and were assessed according to the World SIVA International Sedation Task Force Tool [22] complemented by a previous publication when appropriate [23]. The same observer who accompanied the child throughout the dental sedation procedure registered any adverse event during the intra- or post-operative periods. Then, for up to 24 hours after the appointment, a research team member called the parent to investigate any complications.

There were no changes to the aforementioned outcomes after the trial commenced.

### Sample size

The sample size was confirmed in an interim analysis by a blinded researcher, which included the first 30 children participating in the present study. In this analysis, the success rate observed in the three groups (MO 20%, KMO 30%, and KMIN 60%) was adopted as a parameter. Quiet behavior (OSUBRS 1) from the child for at least 60% of the session length was defined as success. Only the extremes (20% and 60%) were considered in the calculation, allowing a viable number of participants. These analyses confirmed that a sample of at least 28 children in each group would be required.

### Randomization and blinding

A researcher not directly involved in the clinical procedures performed the randomization procedure using an online calculator (www.randomization.com), establishing five blocks with 15 participants each, and the last block with nine participants. The code obtained in the randomization for each consecutive participant was kept in a sealed brown envelope under the strict care of the anesthesiologist. The envelopes were sequentially numbered and opened consecutively by the anesthesiologist soon after the parent signed the consent form agreeing to the child’s participation in the study.

To ensure the blinding, participants (children and parents/caregivers) and the researchers involved in dental treatment, data collection, and statistical analysis, were unaware of which sedative was administered to each child. Only the pediatrician and the anesthesiologist knew the allocation group for each patient so they could prepare the drugs and act immediately if any adverse event occurred. The placebo syrup had the same color and density of the midazolam oral syrup, as did the intranasal placebo (saline), which was administered in the same type of syringe/atomizer used for intranasal ketamine or midazolam. Both placebos were given in the same volume that would be expected for the active drug.

### Statistical analyses

The outcome variable ‘child behavior’ was approached in three formats, to better illustrate the results provided by the three sedative regimes: 1. Dichotomous, according to the quiet behavior for at least 60% of the session length (similar to the format used for sample size calculation); 2. Continuous, represented by the observed percentage of each of the four OSUBRS scores in a session; 3. Ordinal, in which the IBM SPSS 24.0 visual binning tool was employed to determine 3 cut points with an equal number of cases in each bin and then generate four classes of behavior, considering the percentage of the quiet OSUBRS score throughout a dental sedation appointment.

The data were analyzed using the statistical software IBM SPSS 24.0 (IBM Corporation, Armonk, NY, USA), with a significance level of 5%. Analyses included descriptive statistics and association tests for comparisons among the three groups. Kruskal-Wallis and chi-square (Pearson’s or Fisher Exact test) tests with Bonferroni correction were used to verify the association between behavior and the three groups. A subgroup analysis for children’s age (2–3 years old and 4–6 years old) comparing sedative groups regarding children’s behavior was planned. Friedman's test was used to compare vital signs at the baseline and session under sedation. Effect sizes (odds ratio ‘OR’), number needed to treat (NNT) and relative risk reduction were also estimated using the dichotomous behavioral assessment (success/failure of sedation); the MO group was used as control for both KMIN and KMO.

## Results

**Fig 1:**
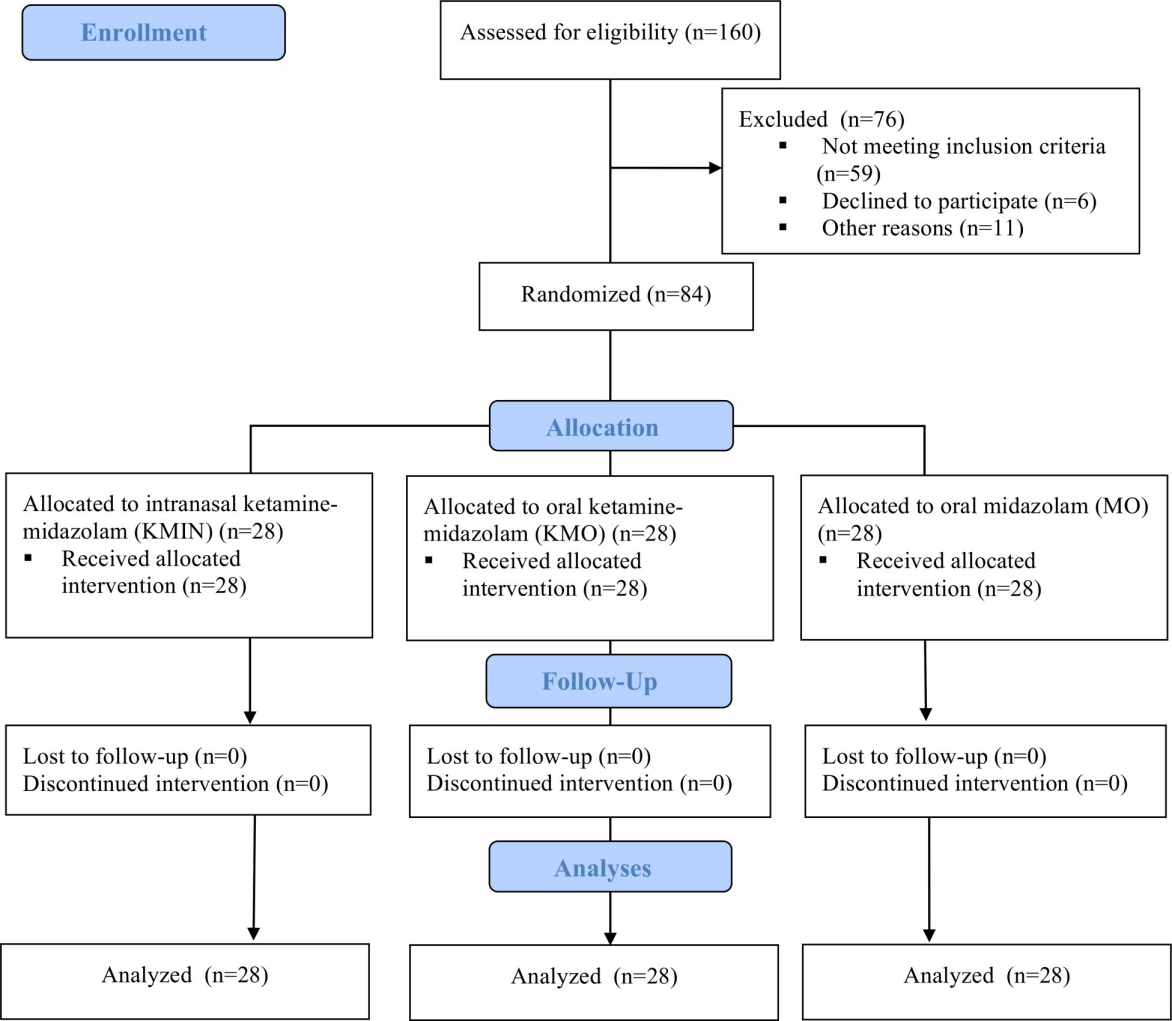
CONSORT flowchart of clinical trial progress stages.

A total of 84 children (43 boys; 51.2%), with a mean age of 3.1 years (SD 1.2), were enrolled in this study between May 2015 and October 2016 (Fig 1). All children were included in the final analyses. Study groups were balanced with regard to demographic data, baseline clinical characteristics, and procedural sedation sessions’ data (Table 1). Dental treatment was completed in 92.9% (KMIN, n = 26), 89.3% (KMO, n = 25) and 85.7% (MO, n = 24).

**Table 1:** Characteristics of participants and dental sedation appointments according to the groups.

Variables	Sedative groups		
	Intranasal ketamine–midazolam (n = 28)	Oral ketamine–midazolam (n = 28)	Oral midazolam (n = 28)
**Children's characteristics**			
Sex^a^			
Female	14 (50.0%)	13 (46.4%)	14 (50.0%)
Male	14 (50.0%)	15 (53.6%)	14 (50.0%)
Age (months)^b^	43.5 (33.0–52.3)	38.0 (33.3–48.0)	42.5 (35.0–47.5)
Weight (kg)^b^	15.7 (13.5–17.5)	14.5 (13.0–17.4)	15.4 (14.2–17.3)
ASA^a^			
I	23 (82.1%)	28 (100.0%)	25 (89.3%)
II	5 (17.9%)	0 (0%)	3 (10.7%)
**Dental history**			
Current toothache^a^	20 (71.4%)	14 (50.0%)	19 (67.9%)
Local anesthesia in previous dental treatment^a^	12 (42.9%)	6 (21.4%)	12 (42.9%)
Number of decayed teeth^b^	9.0 (6.0–11.0)	8.5 (4.0–10.0)	7.0 (6.0–9.0)
Physical restraint during dental exam (recruiting phase)^a^	17 (60.7%)	21 (75.0%)	20 (74.1%)
**Vital signs just before sedative administration**			
Heart rate (beats per minute)^b^	110.0 (98.3–120.0)	102.0 (98.0–113.0)	103.0 (98.3–123.0)
Oxygen saturation (%)^b^	98.0 (96.0–98.8)	98.0 (97.0–98.0)	97.0 (97.0–98.8)
Respiratory rate (breaths per minute)^b^	24.0 (24.0–27.5)	24.0 (24.0–28.0)	24.0 (24.0–25.5)
Blood pressure (mmHg)^b^			
Systolic	90.0 (80.0–90.0)	85.0 (85.0–90.0)	90.0 (85.0–100.0)
Diastolic	60.0 (55.0–60.0)	60.0 (55.0–60.0)	60.0 (60.0–60.0)
**Dental sedation appointment**			
Positive behavior at the entrance to the dental office^a^	23 (82.1%)	26 (96.3%)	21 (77.2%)
Length of the procedure (minutes)^b^	24.0 (21.0–28.8)	27.5 (23.0–35.0)	24.0 (20.5–29.0)
Procedure aborted^a^	2 (7.1%)	3 (10.7%)	4 (14.3%)
Physical restraint needed (except mouth prop)^a^	18 (64.3%)	16 (57.1%)	21 (75.0%)

^a^ n (%)

^b^Median (1^st^-3^rd^ quartiles)

**Fig 2:**
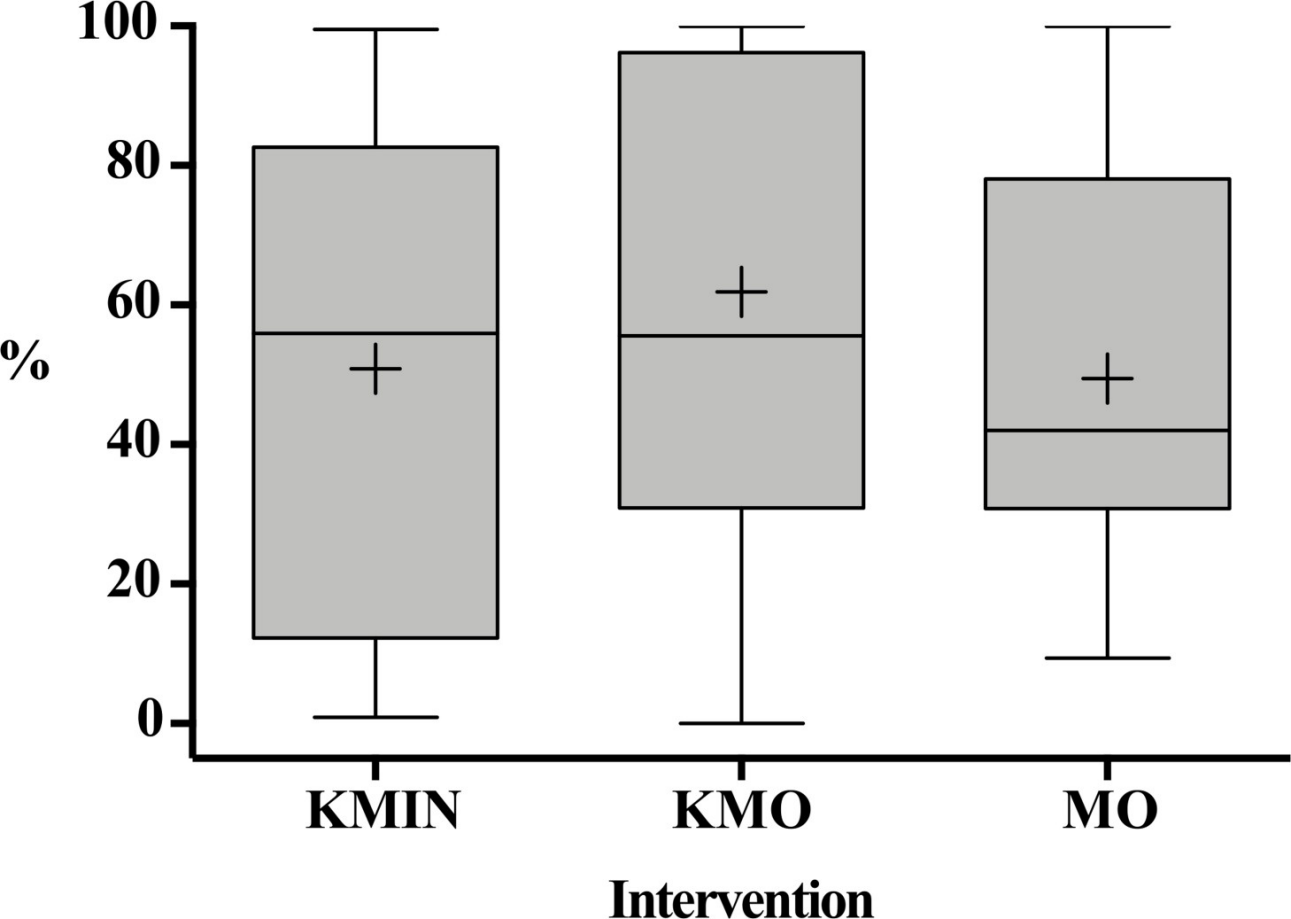
Percentage of quiet behavior in the sedative groups reported as minimum to maximum values, medians, quartiles and means (+). KMIN = Intranasal ketamine–midazolam; KMO = Oral ketamine and midazolam; MO = Oral midazolam.

Overall, the percentage of quiet behavior ranged from 0 to 100% with a substantial variability in the three sedative groups (Fig 2). Medians were higher in the KMIN and KMO groups, but statistically similar to the MO group (P = 0.22, Kruskal-Wallis test); 95% confidence intervals of medians were: KMIN (17%, 80%), KMO (45%, 95%), MO (34%, 69%).

The categories of quiet behavior generated through visual binning comprised 21 cases each one: often quiet (median 96.5% of quiet behavior; quartiles 93.6%, 98.7%); quiet most of the time (70.7%; 56.3%, 81.2%); quiet a few times (36.4%; 32.5%, 44.2%); restless (11.7%; 4.3%, 17.9%). The Chi-square with adjusted p-value through the Bonferroni method did not reveal a difference among groups (Table 2, P = 0.05); pairwise comparisons of column proportions uncovered that KMO presented more 'often quiet behavior' (42.9%) than MO (10.7%), but similar to KMIN (21.4%).

**Table 2:** Children’s behavior under sedation during the dental treatment.

Categories	Intervention groups		
	Intranasal ketamine–midazolam (KMIN) (n = 28)	Oral ketamine–midazolam (KMO) (n = 28)	Oral midazolam (MO) (n = 28)
Restless	10 (35.7%)^a^	5 (17.9%)^a^	6 (21.4%)^a^
Quiet a few times	4 (14.3%)^a^	6 (21.4%)^a^	11 (39.3%)^a^
Quiet most of the time	8 (28.6%)^a^	5 (17.9%)^a^	8 (28.6%)^a^
Often quiet	6 (21.4%)^a^^,^^b^	12 (42.9%)^b^	3 (10.7%)^a^

Pearson Chi-Square with Bonferroni correction, P = 0.05

^a,b^ Different subscript letter denotes a subset of sedative categories whose column proportions differ significantly from each other

The success of the treatment as assessed by the dichotomous variable ‘quiet behavior for at least 60% of the session length’ was: KMIN 50.0% (n = 14; OR 2.10, 95% CI 0.71 to 6.30), KMO 46.4% (n = 13; OR 1.80, 95% CI 0.62 to 5.40), MO 32.1% (n = 9) (Pearson Chi-square 0.360).

The NNT and efficacy (or relative risk reduction) for the sedation success indicated that, on average, 6 (95% CI 2 to infinite) and 7 (95% CI 3 to infinite) children respectively would have to receive KMIN and KMO, instead of MO, for one additional patient to not have a sedation failure during dental sedation. For the relative risk reduction estimation, there was a 56% (95% CI -199% to 19%) reduction of failure in the KMIN group and 45% (95% CI -182% to 26%) in the KMO group.

There were 62 children aged 2–3 years and 22 children 4–6 years old; this polarization of participants at younger ages undermined the analysis of the efficacy of sedatives on the behavior of children in the two age subgroups as originally planned.

Vital signs (heart and respiratory rate, oxygen saturation, and blood pressure) remained within normal limits and did not significantly change during the dental sedation procedure (Friedman test, *P* > 0.05).

A total of 37 (44.0%) children had intra and/or postoperative adverse events (Table 3), which were minor, did not require any specific intervention and did not associate with the sedative group (Pearson chi-square, P = 0.462).

**Table 3:** Absolute and relative frequencies of adverse events related to sedative groups.

Adverse events^a^	Intervention groups		
	Intranasal ketamine–midazolam (KMIN) (n = 28)	Oral ketamine–midazolam (KMO) (n = 28)	Oral midazolam (MO) (n = 28)
**Total**	11 (39.3%)^b^	15 (53.6%)^c^	11 (39.3%)
**Intraoperative**			
Vomit	1 (3.6%)	1 (3.6%)	2 (7.1%)
Prolonged recovery	0	0	3 (10.7%)
**24 hours postoperatively**			
Nausea/vomiting	5 (17.9%)	6 (21.4%)	6 (21.4%)
Irritability	3 (10.7%)	2 (7.1%)	5 (17.9%)
Agitation	1 (3.6%)	5 (17.9%)	2 (7.1%)
Drowsiness	0	2 (7.1%)	1 (3.6%)
Hallucination	1 (3.6%)	2 (7.1%)	1 (3.6%)

^a^ One child could have more than one adverse event

^b^ Odds ratio 1.0 (95% CI 0.34 to 2.90) in contrast to MO

^c^ Odds ratio 1.8 (95% CI 0.62 to 5.20) in contrast to MO

## Discussion

Overall, the combination of ketamine/midazolam produced satisfactory behavior in the majority of children and for most of the procedural sedation appointment. However, the study results refute the hypothesis that the combination of ketamine and midazolam delivered through the intranasal route improves the behavior of children undergoing dental treatment, compared with the same sedative combination delivered through the oral route as well as with the midazolam alone.

The investigated interventions are comparable with one trial that administered ketamine (10 mg/kg) and midazolam (0.5 mg/kg) through oral or intranasal routes in 23 young children [12]. However, that study [12] presents important methodological differences when compared to this study, such as crossover design, combination between the sedation regime and 40% nitrous oxide, and non-trained professionals assessing the children’s behavior. Crossover design is recommended when the underlying condition is stable, and, between intervention moments, there will be no significant changes [24]. This does not occur when we investigate the effect of sedatives on the same child at different times. Children’s behavior may be the result of previous experience, such as negative dental experience [25]. Therefore, if the first trial intervention is unpleasant for the child, this result may affect the success of the subsequent intervention [6]. This prior study [12] may have been affected by biases which limits comparison with this parallel, randomized clinical trial.

We hypothesized that there would be greater occurrence of quiet behavior among sedated children with KMIN because of the advantages associated with the intranasal route, including more reliable sedative absorption than the oral route, since it avoids first pass hepatic metabolism [11,26]. Perhaps a persistent unpleasant perception of the intranasal sedative by the child may have impaired the expected efficacy of the KMIN group. In an attempt to control the nasal discomfort associated with midazolam [17], we first atomized ketamine and waited three minutes to administer midazolam, because that would be enough time to observe an initial local analgesic properties of ketamine [27,28]. However, the intranasal administration of an intravenous formulation of ketamine has a bad taste even if nebulized into the nose [29]. The effect of the intranasal administration on the children’s behavior during a procedural sedation should be further investigated in future studies.

Additionally, as we aimed to deliver moderate sedation, children’s psychological characteristics might have influenced their behavior even during sedation [30–32], but the literature is scarce on this kind of investigation. Ultimately, considering the extreme negative behavior observed in some children while they were sedated, we should speculate that they would be better indicated to general anesthesia [33]. Indeed, there is no uniformity in the policy of indicating sedation or general anesthesia in pediatric dentistry in a global context [34]. These factors may have influenced the variability in the data of the main outcome and consequent loss of power of the sample, although we did an interim analysis with the first 30 cases included in the study to estimate the sample size.

In this trial, the dose of midazolam differed in the KMIN and KMO groups. Midazolam doses of 0.5 mg/kg for the oral route and 0.2 mg/kg for the intranasal route are in agreement with previous studies [17], and supported by the fact that the bioavailability of the nasal route is more than twice of the oral route (75% vs. 36%) [35,36]. Intranasal use of doses greater than 0.2 mg/kg causes excessive coughing and sneezing with greater expulsion of the drug [18], while doses of 0.5 mg/kg of oral midazolam provide good sedation [20]. Nonetheless, KMO improved children’s behavior compared with MO, confirming previous findings [9,10]. In fact, ketamine adds to the procedural sedation in children, given that it can promote a dissociative state, preserves cardiovascular stability [37], does not cause respiratory depression [38], and possesses analgesic effects, which promote reductions in postoperative pain [39].

There is no ideal instrument to evaluate the efficacy of sedation [40]. Children’s behavioral evaluation is one method to assess it and is often performed through observational scales such as the OSUBRS, which provides accurate dynamic behavioral data during sessions [41]. This scale presents four behavioral categories ranging from quiet to combative that generates continuous data. In this study, we added analyzes beyond that used to calculate sample size [14] to better understand the effect of sedative regimens on children's behavior. Preliminarily, we planned to dichotomize the children's behavior in more than 60% of quiet behavior during the session (success of sedation) and less than 60% of quiet behavior during the session (sedation failure) [14]. However, this would hide the variety of in-between behaviors that can be observed in a moderately sedated child. Therefore, we categorized the percentage of quiet behavior into four groups to improve the clinical understanding of our results. Another four-category scale indicated to assess children's behavior during dental treatment corroborates this type of classification [42].

In fact, adding ketamine (in both KMIN and KMO) had the potential to reduce the risk of negative behavior in relative and absolute terms. Although the NNTs obtained in this study were not significant, it can be speculated that ketamine associated with midazolam could be effective compared to midazolam alone; larger sample sizes can elucidate this issue. Completion of treatment is also a way of assessing the success of sedation [6]. In this study, we did complete dental treatments in 75 of a total of 84 sessions despite the need for protective stabilization. Thus, we consider that the sedative regimes investigated were successful in most sessions.

This study has several limitations that should be considered. The shorter times of action and recovery of the intranasal route [43] may explain the fact that there was no better behavior among children sedated via this route, compared to the oral route. In the case of these children, there seems to be less time to perform procedures and, consequently, the possibility of non-cooperative behavior during the procedure. However, these aspects were not evaluated in this study because of the standardization of drug administration time for blinding purposes and subsequent reliability of the measures [44]. The absence of this evaluation does not compromise the results of the research, which is evidenced by the similarity in the number of aborted procedures and in the duration of the consultation. Future clinical studies comparing times of action and recovery among sedatives administered by intranasal and oral routes are needed.

In addition, we partially changed the statistical planning [14], which projected the independent variables: child’s sex, age, dental history and caries index, as well as need for protective stabilization during sedation and length of the procedural session. Actually, the limited sample size and the balance of these variables in the sedation groups did not allow a meaningful logistic regression analysis.

On the other hand, this clinical trial has several strengths that increase its internal validity. We confirmed the uncooperative behavior of children before they were included in the study, because they might have been referred for sedation by dentists with difficulties in the use of non-pharmacological behavioral management techniques. Dental procedures were standardized to reduce the variability of stimuli across groups. Further, necessary training of the observers who assessed the main outcome was performed until there was an inter-examiner agreement of 85%. In addition, one of the measures of children’s behavior was measured continuously, using the OSUBRS scale with specific software, which ensures more realistic and representative data of a procedural sedation session, if compared to more subjective perceptions of the staff. We also warranted that the research team and participants of the research were masked to the intervention group, with the exception of the pediatrician and anesthesiologist, who were able to act in the case of severe adverse events.

The results of this study are useful to guide practitioners in the selection of the appropriate sedative and administration route, while better evidence is not available. We suggest that, in pediatric dentistry, the combination of ketamine and midazolam may be administered via the route more favorable for the patient. Children who have good oral acceptance may benefit from ketamine/midazolam administration via this route. When the oral route is not well accepted, the intranasal route can be chosen. The administration of sedatives should be performed by a competent professional to use such techniques, perform adequate monitoring of the patient and handle the possible intercurrences. As there are safety concerns on the use of ketamine by non-anesthesiologists, it must be administered by qualified physicians who can provide all levels of analgesia/sedation and advanced airway support if necessary [45].

In conclusion, the combination of ketamine with midazolam appears to be more effective in managing the behavior of non-cooperative children during dental treatment in comparison to midazolam alone, although no statistical significance was reported in this study. Future studies with larger sample sizes should be performed to confirm or refute the present results.

## Supporting information

S1 Appendix CONSORT checklist. (DOC)

S1 Fig Sequence and time intervals for sedative administration among the different groups. (PDF)

S1 Protocol Original study protocol in Portuguese, approved by the Research Ethics Board. (PDF)

S2 Protocol Original study protocol, English translation. (PDF)
