# Supplementary material for: Randomized clinical trial on the efficacy of intranasal or oral ketamine-midazolam combinations compared to oral midazolam for outpatient pediatric sedation
Source: PLoS One. 2019 Mar 11;14(3):e0213074. doi: 10.1371/journal.pone.0213074 (PMC6411109; doi:10.1371/journal.pone.0213074)
Supplement: S1 Fig — (PDF) [file pone.0213074.s002.pdf]

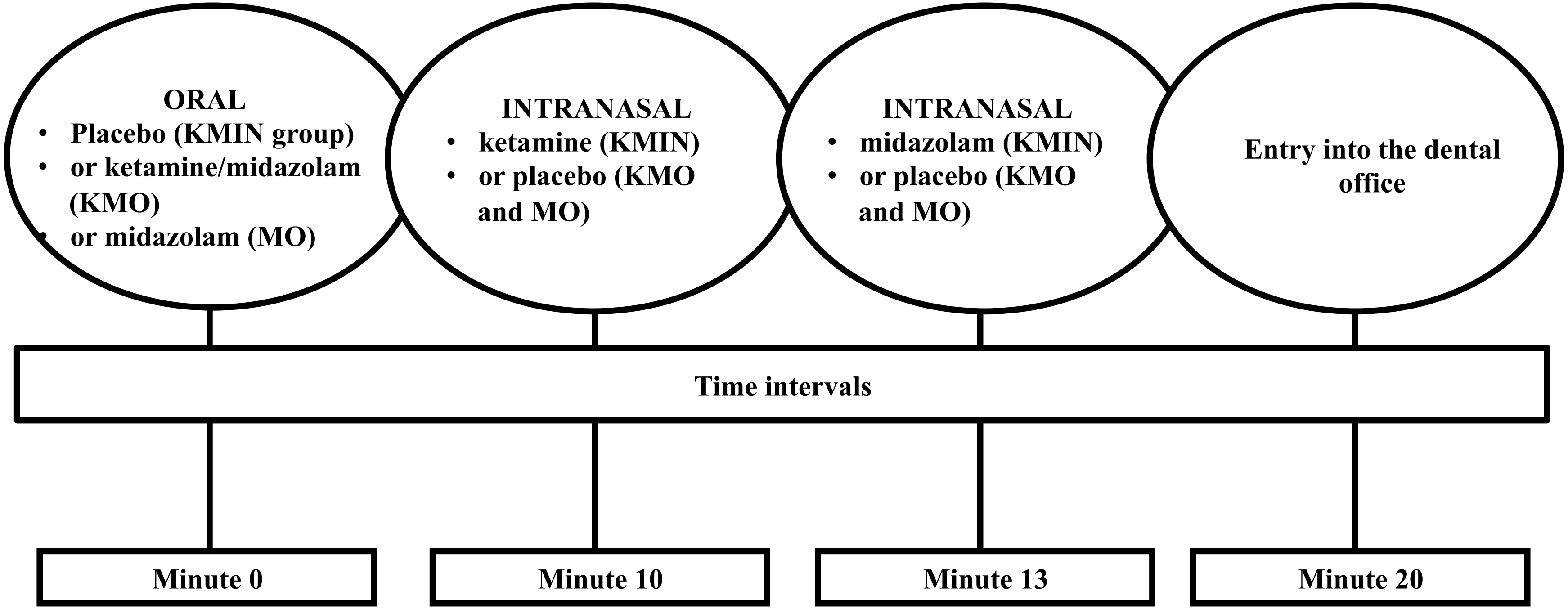

Fig 1. Sequence and time intervals for sedative administration among the different groups. KMIN = Intranasal ketamine and midazolam; KMO = oral ketamine and midazolam; MO = oral midazolam.
