# Supplementary material for: Randomized clinical trial on the efficacy of intranasal or oral ketamine-midazolam combinations compared to oral midazolam for outpatient pediatric sedation
Source: PLoS One. 2019 Mar 11;14(3):e0213074. doi: 10.1371/journal.pone.0213074 (PMC6411109; doi:10.1371/journal.pone.0213074)
Supplement: S1 Protocol — (PDF) [file pone.0213074.s003.pdf]

Projeto de Pesquisa:
Eficácia, segurança e custo-efetividade da sedação intranasal com cetamina e midazolam em odontopediatria: ensaio clínico randomizado

Informações Preliminares

Responsável Principal

CPF/Documento: 510.126.341-91	Nome: Luciane Ribeiro de Rezende Sucasas da Costa
Telefone: 6232096325	E-mail: lsucasas@ufg.br

Instituição Proponente

CNPJ:	Nome da Instituição: Faculdade de Odontologia da Universidade Federal de Goiás

Essa submissão de emenda é exclusiva do seu Centro Coordenador?

A emenda é exclusiva de seu Centro Coordenador, então as alterações realizadas em seu projeto, em virtude da emenda, NÃO serão replicadas nos Centros Participantes vinculados e nos Comitês de Ética das Instituições Coparticipantes, quando da sua aprovação.

É um estudo internacional? Não

Equipe de Pesquisa

CPF/Documento	Nome
284.341.228-55	Aline Carvalho Batista
042.947.331-10	Kárita Cristina Silva
480.068.231-20	Geovanna de Castro Morais Machado
180.922.628-70	Liliani Aires CAndido Vieira
019.437.701-66	JOJI SADO FILHO
024.142.381-35	Karolline Alves Viana
025.787.211-65	Anna Alice Anabuki
081.855.646-39	Heloisa de Sousa Gomes
802.184.901-00	Anelise Daher Vaz Castro
383.187.171-04	Paulo Sérgio Sucasas da Costa
084.368.856-43	Patrícia Corrêa de Faria
252.503.291-87	Mara Rúbia de Camargo Alves Orsini
036.674.591-30	Mônica Maia Moterane

Área de Estudo

Grandes Áreas do Conhecimento (CNPq)

- Grande Área 4. Ciências da Saúde

Propósito Principal do Estudo (OMS)

- Clínico

Título Público da Pesquisa: Eficácia, segurança e custo-efetividade da sedação intranasal com cetamina e midazolam em odontopediatria: ensaio clínico randomizado

Expansão do Acrônimo do Público: Estudo da administração Nasal de sedativos em Odontologia

Contato Público

CPF/Documento	Nome	Telefone	E-mail
510.126.341-91	Luciane Ribeiro de Rezende Sucasas da Costa	6232096325	lsucasas@ufg.br

Contato Científico: Luciane Ribeiro de Rezende Sucasas da Costa

Desenho de Estudo / Apoio Financeiro

Desenho do Estudo: Intervenção/Experimental

Condições de saúde ou problemas

Condição de saúde ou Problema
Ansiedade odontológica
Problema de comportamento em odontologia
Cuidado odontológico para crianças
Cárie na primeira infância

Descritores Gerais para as Condições de Saúde

CID1-10:Classificação Internacional de Doenças

Código CID	Descrição CID
R52.0	Dor aguda
F41.1	Ansiedade generalizada

DeCS:Descritores em Ciência da Saúde

Código DECS	Descrição DECS
E03.250	Sedação consciente
E06.170.152	Assistência Odontológica para Crianças

Descritores Específicos para as Condições de Saúde

CID1-10:Classificação Internacional de Doenças

Código CID	Descrição CID
K02	Carie dentaria

DeCS:Descritores em Ciência da Saúde

Código DECS	Descrição DECS
F01.470.132.300	Ansiedade ao Tratamento Odontológico

Tipo de Intervenção: Comparador

Natureza da Intervenção

- Fármaco/Medicamento/Vacina

Descritores da Intervenção

Descritores da Intervenção

Intervenções
Administração intranasal de cetamina e midazolam
Administração oral de cetamina e midazolam
Administração oral de midazolam
Midazolam

Lista de CID

Código CID	Descrição CID
F41.1	Ansiedade generalizada

Lista de DECS

Código DECS	Descrição DECS
E03.250	Sedação consciente

Fase

- Fase 4

Haverá uso de placebo ou a existência de grupos que não serão submetidos em nenhuma intervenção:

Haverá uso de placebo apenas para comparar as vias de administração intranasal e oral, porém todas as crianças receberão sedativo para tratamento odontológico. Ou seja, quando a criança receber sedativo por via intranasal, receberá placebo por via oral, e vice-versa.

Desenho:

Ensaio clínico randomizado com mascaramento e delineamento paralelo

Apoio Financeiro

CNPJ	Nome	E-mail	Telefone	Tipo
				Financiamento Próprio
01.263.896/0004-07	MINISTERIO DA CIENCIA, TECNOLOGIA E INOVACAO		2121232703	Institucional Principal

Palavra Chave

Palavra-chave
Midazolam
Sedação consciente
Odontopediatria
Cetamina

Detalhamento do Estudo

Resumo:

Cerca de 10% das crianças pode apresentar problema de comportamento durante tratamento odontológico, mas as evidências para protocolos sedativos que beneficiem a assistência em odontopediatria ainda são fracas. Neste ensaio clínico randomizado, mascarado e controlado, de delineamento paralelo, pretende-se avaliar a eficácia, segurança e custo-efetividade da sedação intranasal com cetamina/midazolam em crianças saudáveis de 2 a 6 anos de idade. As crianças serão selecionadas dentre aquelas com cárie dentária que não permitirem atendimento em duas consultas, e necessitam de pelo menos de duas restaurações sob anestesia local e isolamento absoluto. Calcula-se preliminarmente uma amostra de 84 crianças distribuídas igualmente em três grupos: A) cetamina (4,0 mg/kg, máximo 100 mg) + midazolam (0,2 mg/kg, máximo 5 mg) por via intranasal; B) cetamina (4,0 mg/kg, máximo 100 mg) + midazolam (0,5 mg/kg, máximo 20 mg) por via oral; C) midazolam (1,0 mg/kg, máximo 20 mg) por via oral. A hipótese é que associação cetamina/midazolam, administrada por via intranasal, constitui-se em regime sedativo eficaz, seguro e custo-efetivo para uso em odontopediatria no serviço público. O desfecho primário é o comportamento da criança avaliado minuto a minuto, segundo escala de Houpt, nos arquivos dos filmes das sessões de sedação. Os desfechos secundários são: aceitação da administração do sedativo, memória do procedimento e nível de cortisol salivar, dentro de cada grupo de intervenção. Adicionalmente, serão avaliados: ocorrência de dor e associação de alterações nas expressões faciais com estímulos potencialmente dolorosos em crianças sedadas durante o atendimento odontológico; percepção dos acompanhantes (n=84) e do dentista (n=6) sobre a sedação, por meio de auto-relato e estresse medido pelo cortisol salivar; percepção da criança acerca do tratamento odontológico sob sedação, por meio de teste projetivo; eventos adversos ocorridos durante e após a administração dos sedativos; custo-efetividade dos diferentes protocolos sedativos. O delineamento experimental proposto visa evitar erros sistemáticos e aleatórios, para contribuir com maior nível de evidência em revisões sistemáticas futuras. Os desfechos deste estudo apresentam impacto potencial na prática em saúde, pública e privada, e poderão servir para orientação de condutas em diretrizes institucionais vinculadas ao Sistema Único de Saúde e entidades formadoras de opinião.

Introdução:

2.1 Pergunta de pesquisaQual a eficácia, segurança e custo da sedação intranasal com cetamina/midazolam para crianças com problema de comportamento durante o tratamento odontológico?2.2 RelevânciaOs problemas de comportamento durante o tratamento odontológico de crianças e adolescentes afligem cerca de 9% da população mundial. Embora diminuam com a idade e sejam mais observados em meninas, esses problemas de comportamento são associados a medos gerais, problemas comportamentais externalizantes e internalizantes (Klingberg et al., 2007).Tradicionalmente, a abordagem da criança não cooperativa na cadeira do dentista é feita por métodos não farmacológicos (contenção física) ou farmacológicos (sedação ou anestesia geral), associados a técnicas comunicativas que encorajem o enfrentamento da situação pela criança. A tomada de decisão para a escolha do tipo de abordagem depende de: necessidade odontológica, estágio de desenvolvimento da criança, formação do dentista, recursos físicos e humanos disponíveis e escolha dos pais ou responsáveis pela criança.A contenção física, modernamente denominada estabilização protetora, pode ser indicada em situações de necessidade de conduta imediata e, de preferência, associada a um sedativo, para evitar o trauma psicológico à criança (American Academy of Pediatric Dentistry “AAPD”, 2013-2014). A contraindicação ao uso rotineiro da contenção física deve-se ao fato de esta poder se constituir em uma forma de violência contra a criança. Uma das recomendações do Plano Nacional pela Primeira Infância (2010, p. 68) é o“Reconhecimento dos castigos físicos e humilhantes como formas de violência contra a criança, sendo, portanto, uma violação aos seus direitos fundamentais com impacto no desenvolvimento infantil saudável.”A sedação visa acalmar o paciente, promover sono e/ou aumentar o limiar da dor. Ainda, causa depressão da consciência em diferentes níveis – mínima, moderada, profunda. A maioria dos sedativos tem o potencial de causar amnésia, o que favorece o impacto nas reações da criança após um tratamento em que o sedativo não foi efetivo. A sedação moderada tem sido a mais indicada para crianças pré-cooperativas ou com ansiedade leve, desde que a necessidade odontológica não exija grande número de sessões para conclusão de tratamento. Nesse sentido, a taxa de sucesso das sedações em odontopediatria é variável, altamente dependente do regime sedativo (quadro 1). Desde que realizada sob a égide de um protocolo bem estabelecido, a ocorrência de eventos adversos associados à sedação é baixa (quadro 1).A anestesia geral é o método indicado para crianças muito jovens, incapazes de cooperar até sob efeito de sedação, e que apresentem extensa necessidade de tratamento odontológico (AAPD 2013-2014). Porém, devido às maiores exigências para realização de anestesia geral, esta acaba se tornando um procedimento mais oneroso do ponto de vista de recursos humanos, estruturais e financeiros, o que justifica a busca por alternativas eficazes para o gerenciamento comportamental de crianças com problemas de comportamento durante o tratamento odontológico A cárie dentária ainda é a maior causa de necessidade de tratamento odontológico em crianças e, apesar de ter sua prevalência diminuída em alguns países, é uma doença que ainda acomete milhares de pessoas em todo o mundo. No Brasil, apesar dos significativos investimentos do governo e do declínio nos índices de prevalência, 53,4% das crianças brasileiras tem cárie na dentição decídua. Aos cinco anos de idade, uma criança brasileira possui, em média, 2,43 dentes com experiência de cárie, com predomínio do componente cariado, que é responsável por mais de 80% do índice (Brasil, 2010).2.3 JustificativaBases de dados de artigos científicos mostram que muitos pesquisadores e clínicos preocupam-se em identificar o protocolo sedativo que promova o maior conforto possível no tratamento odontopediátrico, com menor risco. Segundo a base de dados PubMed (palavras-chave dental, sedation, child), há 785 artigos publicados desde 1965 até 07/06/2014 sobre esse assunto. No entanto, quando essa busca se restringe a ensaios clínicos controlados aleatorizados (RCT), o número cai para 117 artigos.Lourenço-Matharu et al. realizaram uma revisão sistemática na base Cochrane, publicada em 2012, com o objetivo de avaliar a eficácia de agentes e doses na sedação moderada visando o gerenciamento comportamental em odontopediatria. Identificaram 36 estudos

publicados até 04/11/2011, os quais continham vieses. Concluíram que há fraca evidência de que o midazolam oral é um agente sedativo eficaz para crianças submetidas a tratamento odontológico, e evidência muito fraca em favor do óxido nitroso. Os autores constataram também que é necessário conduzir ensaios clínicos com delineamento adequado para avaliar outros potenciais agentes de sedação, e que se avalie regimes experimentais em comparação com midazolam por via oral ou sedação inalatória com óxido nitroso.Desde então, 10 RCT sobre sedação em odontopediatria foram publicados, investigando diferentes protocolos para distintas faixas etárias (Quadro 1). No entanto, grande parte deles ainda comete inadequações metodológicas como: delineamento cruzado; avaliações de desfechos por examinadores não treinados/calibrados; carência de análises sobre dor, estresse, memória, custo do procedimento sob sedação, e outros aspectos da percepção dos participantes (criança/acompanhante) sobre o sucesso da sedação.Dessa forma, justifica-se a releitura da sedação em odontopediatria por meio de RCT que busque avaliar diferentes indicadores relacionados ao sucesso da sedação. Nosso grupo de pesquisa tem obtido bons resultados com a combinação cetamina/midazolam (Moreira et al., 2013), consequente a um histórico de estudos com outras drogas. Como o uso intranasal de medicamentos parece resultar em melhores resultados do que o oral, o foco da presente pesquisa será investigar a associação cetamina/midazolam por essa via.

Hipótese:

A hipótese é que associação cetamina/midazolam, administrada por via intranasal, constitui-se em regime sedativo eficaz, seguro e custo-efetivo para uso em odontopediatria no serviço público.

Objetivo Primário:

Investigar regimes sedativos com cetamina e midazolam que beneficiem o tratamento odontológico de crianças pré-escolares.

Objetivo Secundário:

Comparar a eficácia da administração intranasal de cetamina/midazolam à administração oral de cetamina/midazolam e de midazolam em crianças de 2 a 6 anos de idade, por meio dos seguintes indicadores:
- Aceitação da administração do sedativo.
- Comportamento das crianças durante o procedimento odontológico.
- Memória do procedimento.
- Nível de cortisol salivar.Avaliar a ocorrência de dor e a associação de alterações nas expressões faciais com estímulos potencialmente dolorosos em crianças sedadas durante o atendimento odontológico.

Buscar a percepção dos acompanhantes e do dentista sobre a sedação, por meio de auto-relato e estresse medido pelo cortisol salivar.

Analisar a percepção da criança acerca do tratamento odontológico sob sedação.

Detectar os eventos adversos ocorridos durante e após a administração dos sedativos.

Realizar análise de custo-efetividade dos diferentes regimes sedativos.

Verificar o impacto da cárie dentária e fatores associados, bem como da sedação odontológica, na percepção dos acompanhantes sobre a qualidade de vida relacionada à saúde bucal (QVRSB) de pré-escolares não cooperativos com o tratamento odontológico.

Metodologia Proposta:

4.1 TIPO DE ESTUDO: Ensaio clínico randomizado, mascarado e controlado, de delineamento paralelo. Nesta emenda, considerando-se o objetivo específico citado no item 1, observa-se a necessidade de um delineamento longitudinal prospectivo. Para isso, as crianças incluídas no ensaio clínico e seus responsáveis ou acompanhantes serão submetidos a avaliações periódicas para verificar mudanças na QVRSB. As avaliações serão realizadas em três momentos, sendo o primeiro (baseline- T0) anterior ao tratamento e as demais no período de duas semanas e três meses após a conclusão do tratamento odontológico. A partir dos escores obtidos no baseline, a amostra será dividida em dois grupos, por meio da análise de cluster, de acordo com maior ou menor escore observado no instrumento B-ECOHIS. Essa análise será realizada após a obtenção dos dados iniciais de toda a amostra.
4.2 LOCAL DO ESTUDO:Este estudo será realizado na Faculdade de Odontologia da Universidade Federal de Goiás (FO/UFG), na estrutura do projeto de extensão “Núcleo de Estudos em Sedação Odontológica” (NESO). O NESO que possui a infraestrutura necessária para realização de tratamento odontológico sob sedação, a saber: equipamento odontológico completo; monitores de frequência cardíaca, saturação de oxigênio (oxímetro de pulso), de dióxido de carbono (capnografia) e de pressão arterial; sedativos e respectivas drogas antagonistas; material para administração de medicamentos por via oral e endovenosa; material para intubação orotraqueal; cilindro de oxigênio; carrinho de anestesia; kit de drogas de emergência (exemplos: adrenalina, hidrocortisona, diazepam, glicose, atropina etc.). Além disso, o NESO integra uma equipe multidisciplinar treinada e capacitada para atuar em sedação odontológica: cirurgião-dentista, médico pediatra, anestesiologista, odontopediatra, psicóloga, cirurgião bucomaxilofacial. Em termos de saúde pública, o NESO é único no país. Sobretudo, historicamente o NESO tem viabilizado a execução de pesquisas em nível de graduação e pós-graduação que tem alcançado publicações de impacto na área.Dessa forma, o NESO proporciona um ambiente seguro à realização de pesquisas voltadas à sedação de crianças.4.3 ASPECTOS ÉTICOSEsta pesquisa seguirá os preceitos da Resolução CNS/MS 466/2012. Assim que aprovado pelo Comitê de Ética em Pesquisa, o projeto será registrado em uma base de dados de ensaio clínico e a coleta de dados será iniciada. Demais considerações estão descritas no projeto.4.4 PARTICIPANTESPopulação do estudo: crianças de 2 a 6 anos de idade com cárie dentária, encaminhadas com história de não cooperação com atendimento odontológico.Amostragem: não probabilística – amostra de conveniência de crianças encaminhadas de serviços públicos de Goiânia-GO e região.Recrutamento: folhetos (Apêndice A) com informação sucinta sobre a pesquisa serão distribuídos nos postos de saúde e centros de especialidade odontológica, orientando os profissionais de saúde a encaminharem os potenciais participantes da pesquisa para o NESO.4.5 INTERVENÇÕESEste ensaio clínico irá comparar o efeito de três tipos de intervenção (quadro 2). Dessa forma, todas as crianças receberão um regime sedativo, seja por via intranasal ou oral. O grupo A, experimental, ainda carece de resultados na literatura e justifica a presente investigação. Os grupos B e C constituem-se em regimes sedativos que tem avaliações positivas na literatura e servirão para comparação do regime experimental. Apenas o médico saberá o grupo para o qual a criança foi sorteada, em virtude da necessidade de tomar providências imediatas em caso de evento adverso. Assim, a criança/acompanhante, dentista operador e auxiliar e demais profissionais observadores serão mascarados para o grupo de intervenção.4.7 PROCEDIMENTOS DE COLETA DE DADOS4.Por meio de consultas odontológicas, aplicação de questionários e entrevistas.

Critério de Inclusão:

Critérios de inclusão: crianças cujo estado físico seja categorizado como ASA 1 (saudáveis) ou 2 (doença sistêmica leve e controlada – asma persistente, por exemplo); crianças com pequeno risco de obstrução de vias aéreas (Malampati menor que 2 e/ou hipertrofia de tonsilas ocupando menos de 50% da orofaringe); ausência de história médica de alterações neurológicas ou cognitivas; ausência de deformidades faciais; nascimento a termo; crianças que não fazem uso de medicamentos que possam comprometer as funções cognitivas; crianças com pelo menos dois dentes com cárie sem envolvimento pulpar, necessitando restauração dentária sob anestesia local e isolamento absoluto.

Critério de Exclusão:

Critérios de exclusão: comportamento positivo ou definitivamente positivo (Frankl et al., 1962) em uma sessão de tratamento

odontológico realizada pela equipe, pós-treinamento e calibração.

Riscos:

Previamente à coleta dos dados, os responsáveis legais das crianças ou acompanhantes serão esclarecidos, pelo pesquisador, sobre os objetivos da pesquisa, riscos e benefícios. Os riscos e desconfortos esperados aos participantes da pesquisa (criança) são os inerentes ao tratamento odontológico sob sedação. Os eventos adversos relacionados à sedação no NESO são pouco frequentes e de pequena gravidade (Costa et al., 2012). Poderão ocorrer eventos adversos durante o procedimento ou nas primeiras 4 horas após a administração dos sedativos. Esses eventos adversos podem ser de risco mínimo, risco baixo ou risco alto/sentinela (Quadro 2). Caso ocorram, serão adotados os procedimentos constantes no consenso da International Sedation Task Force (ISTF) of the World Society of Intravenous Anaesthesia (World SIVA) (Quadro 2). O primeiro atendimento será realizado no próprio NESO e, se necessário, um membro da equipe acompanhará o paciente até o pronto-socorro do Hospital das Clínicas da UFG, conforme parceria firmada (Apêndice D). Embora sejam raros eventos adversos em domicílio, os responsáveis serão orientados verbalmente e por escrito sobre as recomendações pós-operatórias (Apêndice E), enfatizando a necessidade de entrarem em contato com a equipe em caso de dúvidas. Se necessário, a equipe irá à residência do paciente para avaliá-lo ou, em casos mais graves, acionará o Serviço de Emergência (192) e acompanhará todo o atendimento do paciente. Se ocorrerem eventos adversos em algum momento da pesquisa, será avaliada a necessidade de adequar ou suspender o estudo. Todavia, os recursos e protocolos adotados no NESO visam minimizar a ocorrência de eventos adversos maiores pois são baseados em evidências científicas e aplicam os princípios éticos da beneficência, não-maleficência e alteridade.Os riscos para acompanhante e dentista, que terão sua saliva coletada e responderão a duas perguntas em questionário, serão de quebra de privacidade na coleta das informações ou confidencialidade na divulgação dos resultados, mas a equipe de pesquisa tomará as precauções para evitar que isso aconteça: codificar com senha o acesso aos bancos de dados, colocar o código e não o nome do participante na coluna de identificação dos casos no banco de dados.Com o objetivo específico que foi acrescentado nesta emenda, os riscos do estudo estão relacionados ao possível constrangimento dos responsáveis ou acompanhantes ao responder o questionário. O risco de constrangimento será minimizado proporcionando aos responsáveis ou acompanhantes um ambiente propício para a aplicação do questionário, total liberdade de resposta e questionamentos, além de garantia de sigilo do seu nome e imagem.

Benefícios:

Os benefícios serão diretos e indiretos. A criança será diretamente beneficiada por meio da conclusão do tratamento odontológico e posterior acompanhamento da sua condição bucal. A comunidade científica será beneficiada a partir das evidências produzidas no estudo que deverão ser aplicadas a populações semelhantes à amostra avaliada. Os resultados desta pesquisa têm o potencial de beneficiar outras crianças que se assemelhem às condições desta amostra, na medida em que um regime sedativo eficiente for identificado. Os resultados deste estudo serão publicados, sejam eles favoráveis ou não, e contribuirão com o conhecimento científico atualmente disponível. Os responsáveis ou acompanhantes poderão optar pela desistência de participação no estudo sem que haja prejuízos na sua relação e na da criança com a instituição e a equipe de pesquisa. Essas informações, assim como a descrição da pesquisa estão contidas no Termo de Consentimento Livre Esclarecido (TCLE) que será assinado pelos responsáveis ou acompanhantes em caso de concordância a sua participação e a da criança no estudo (APÊNDICES A, B, C).

Metodologia de Análise de Dados:

4.8.1 Plano amostras: O tamanho da amostra foi calculado com base na variável primária, ou seja, comportamento de crianças durante a sedação. No estudo de Moreira et al. (2013), verificou-se os escores de comportamento medidos pela escala OSUBRS nos grupos midazolam/cetamina (média 8,6; desvio padrão 4,1), midazolam (14,0; 3,8), ambos administrados por via oral, e nenhum sedativo (12,5; 5,2). Com base nesses valores, calculou-se em 23 casos por grupo, comparando os grupos midazolam/cetamina e nenhum sedativo, para se obter um poder do teste de 80% em nível de 5%.Pensando em perda de 20%, estima-se uma amostra preliminar de 28 crianças por grupo. No entanto, como não foi encontrado nenhum ensaio clínico com desenho paralelo que tenha feito as mesmas comparações deste projeto, esse cálculo será confirmado por meio de estudo piloto a ser realizado com o primeiro bloco de casos randomizados.4.8.2 Análise dos dadosAnálises descritivas e bivariadas serão realizadas nos softwares estatísticos Prisma GraphPad e IBM SPSS, considerando o nível de significância de 5%. Após confirmação da distribuição das variáveis contínuas (normal/não normal), os três grupos serão comparados conforme as diferentes variáveis de desfecho observadas na primeira consulta de intervenção (quadro 4).

Desfecho Primário:

A medida de desfecho primária será o comportamento da criança durante o tratamento sob sedação, mas haverá medidas de desfecho secundárias que contribuirão para a compreensão mais abrangente do sucesso da sedação.

Desfecho Secundário:

Aceitação da administração do sedativo.Dor durante tratamento.Memória.Percepção do acompanhante e do dentista sobre sedação.Percepção da criança sobre a sedação.Eventos adversos.

Tamanho da Amostra no Brasil: 174

Países de Recrutamento

País de Origem do Estudo	País	Nº de participantes da pesquisa
Sim	BRASIL	174

Outras Informações

Haverá uso de fontes secundárias de dados (prontuários, dados demográficos, etc)?

Não

Informe o número de indivíduos abordados pessoalmente, recrutados, ou que sofrerão algum tipo de intervenção neste centro de pesquisa:

174

Grupos em que serão divididos os participantes da pesquisa neste centro

ID Grupo	Nº de Indivíduos	Intervenções a serem realizadas
Mães/acompanhantes	84	Responder questionários sobre qualidade de vida; ter saliva coletada para avaliação do cortisol salivar
Grupo A	28	Crianças - sedação intranasal (cetamina/midazolam) + placebo oral; acompanhantes - responderão a questionário e coleta de saliva
Grupo B	28	Crianças - sedação intranasal placebo + sedação oral (midazolam/cetamina); acompanhantes - responderão a questionário e coleta de saliva
Grupo C	28	Crianças - sedação intranasal placebo + sedação oral (midazolam); acompanhantes - responderão a questionário e coleta de saliva
Dentistas	6	Responderão a questionário e terão saliva coletada

O Estudo é Multicêntrico no Brasil?

Não

Propõe dispensa do TCLE?

Sim

Justificativa:

Os pesquisadores solicitam a dispensa do assentimento livre e esclarecido para essa etapa da pesquisa. A dispensa é necessária pelo fato de que as crianças que participarão do estudo têm comportamento não colaborador no atendimento odontológico e isso poderia resultar em grande proporção de recusa em participar. Além disso, outra limitação da aplicação do termo de assentimento é a dificuldade de compreensão dos objetivos da pesquisa por crianças que ainda apresentam-se em fase de desenvolvimento cognitivo. Os pesquisadores, reconhecendo que esta pesquisa envolverá seres humanos vulneráveis, se comprometem a seguir o preceito ético de autonomia da criança, respeitando sua vontade de participar da pesquisa e garantir sua privacidade. Serão garantidos também a integridade física, psíquica e moral destes participantes, conforme estabelecido no Estatuto da Criança e do Adolescente.

Haverá retenção de amostras para armazenamento em banco?

Não

Cronograma de Execução

Identificação da Etapa	Início (DD/MM/AAAA)	Término (DD/MM/AAAA)
Cadastro em base de dados de ensaio clínico	17/11/2014	21/11/2014
Acompanhamento (avaliações pós-tratamento concluido)	17/04/2017	31/12/2017
Pesquisa bibliográfica	01/04/2018	31/12/2019
Análise das amostras de saliva	02/02/2015	31/03/2017
Realização das consultas odontológicas	01/04/2017	30/11/2017
Treinamento e calibrarão da equipe	06/10/2014	14/11/2014
Coleta de dados	01/12/2014	16/12/2016
Recrutamento	24/11/2014	31/08/2016
Produção de documentos científicos: resumos, dissertações, teses e relatórios	01/04/2018	31/12/2019
Análise dos dados	01/04/2018	31/12/2019
Observação dos videos	02/02/2015	31/03/2017
Realização das intervenções	01/12/2014	16/12/2016

Orçamento Financeiro

Identificação de Orçamento	Tipo	Valor em Reais (R$)
Discos rígidos externos para armazenamento de dados da pesquisa	Capital	R$ 1.000,00
Total em R$		R$ 1.000,00

Tablet	Capital	R$ 950,00
Medicamentos relacionados à sedação (incluindo solução placebo)	Custeio	R$ 2.000,00
Câmeras de video digital portátil (2 unidades)	Capital	R$ 3.000,00
Gravador de áudio digital	Capital	R$ 250,00
Vale-transporte para os responsáveis	Custeio	R$ 1.500,00
Material de escritório	Custeio	R$ 2.000,00
Total em R$		R$ 10.700,00

Bibliografia:

American Academy on Pediatric Dentistry Clinical Affairs Committee-Behavior Management Subcommittee. Guideline on behavior guidance for the pediatric dental patient. Pediatr Dent. 2013-2014;35(6):176-87. American Academy of Pediatrics; American Academy on Pediatric Dentistry. Guideline for monitoring and management of pediatric patients during and after sedation for diagnostic and therapeutic procedures. Pediatr Dent. 2013-2014;35(6):205-21. Ashraf AB, Lucey S, Cohn JF, Chen T, Ambadar Z, Prkachin KM, Solomon PE. The painful face – pain expression recognition using active appearance models. Image Vis Comput. 2009;27(12):1788-1796. Bahetwar SK, Pandey RK, Saksena AK, Chandra G. A comparative evaluation of intranasal midazolam, ketamine and their combination for sedation of young uncooperative pediatric dental patients: a triple blind randomized crossover trial. J ClinPediatr Dent. 2011;35(4):415-20. Bhatnagar S, Das U M, Bhatnagar G. Comparison of oral midazolam with oral tramadol, triclofos and zolpidem in the sedation of pediatric dental patients: An in vivo study. J Indian SocPedodPrev Dent 2012;30:109-14. Birnie KA, Noel M, Chambers CT, von Bayer CL, Fernandez CV. The cold pressor task: is it an ethically acceptable pain research method in children? J PediatrPsychol 2011;13:1071-81. Birnie KA, Petter M, Boerner KE, Noel M, Chambers CT. Contemporary use of the cold pressor task in pediatric pain research: a systematic review of methods. J Pain 2012;13:817-26. Boerner KE, Chambers CT, Craig KD, Pillai Riddell RR, Parker JA. Caregiver accuracy in detecting deception in facial expressions of pain in children. Pain. 2013;154(4):525-533. Brasil. Ministério da Saúde, Departamento de Atenção Básica (DAB). Projeto SBBrasil 2010: Pesquisa Nacional de Saúde Bucal: resultados principais. Brasília: Ministério da Saúde, 2011. Brasil. Ministério da Saúde. Secretaria-Executiva. Área de Economia da Saúde e Desenvolvimento. Avaliação de tecnologias em saúde: ferramentas para a gestão do SUS / Ministério da Saúde, Secretaria-Executiva, Área de Economia da Saúde e Desenvolvimento. – Brasília: Ministério da Saúde, 2009. Buffett-Jerrott SE, Stewart SH, Finley GA, Loughlan HL. Effects of benzodiazepines on explicit memory in a paediatric surgery setting. Psychopharmacology (Berl). 2003 Aug;168(4):377-86. Capovilla FC, Negrão V, Damázio M, Roberto MR, Marins KC, Sousa-Sousa CC, Lima-Sousa A, Botelho F. Pictografia evocadora de fala: CD2: BFI-Livres: 3100 figuras com normatização de nomeação do maternal ao ensino superior, e do grau de familiaridade do nome no ensino fundamental. 1. ed. São Paulo, SP: Memnon, 2011. v. 1. Chambers CT, Cassidy KL, McGrath PJ, Gilbert CA, Craig KD. Child Facial Coding System Manual. Halifax, Nova Scotia: Dalhousie University and University of British Columbia, 1996. Chang J, Versloot J, Fashler SR, McCrystal KN, Craig KD. Pain assessment in children: validity of facial expression items in observational pain sclaes. Clin J Pain. 2014 [Epub ahead of print]. Chopra R, Mittal M, Bansal K, Chaudhuri P. Buccal midazolam spray as an alternative to intranasal route for conscious sedation in pediatric dentistry. J ClinPediatrDent. 2013;38(2):171-3. Costa LR, Costa PS, Brasileiro SV, Bendo CB, Viegas CM, Paiva SM. Post-discharge adverse events following pediatric sedation with high doses of oral medication. J Pediatr. 2012 May;160(5):807-13. Didonet V. Plano Nacional pela Primeira Infância. Brasília: Rede Nacional Primeira Infância, 2010. Disponível em: http://primeirainfancia.org.br/wp-content/uploads/PPNI-resumido.pdf>. Acesso em 07 jun. 2014. Cunha JA. Desenvolvimentos do Procedimento de Desenhos-Estórias (D-E). In: CUNHA, Jurema Alcides. Psicodiagnóstico-V. 5. ed. São Paulo: Artmed, 2000. Cap. 29, p. 248-249. Drummond MF, O'Brien B, Stoddart GL, Torrance GW: Methods for the economic evaluation of health care programmes. 3rd ed. New York: Oxford University Press; 2005. Frankl S, Shiere F, Fogels H. Should the parent remain with the child in the dental operatory. J Dent Child. 1962;29:150-163. Freitas NK. CAT e sua interpretação dinâmica. In: Cunha JA. Psicodiagnóstico-V. 5. ed. São Paulo: Artmed, 2000. Cap. 27, p. 416-420. Ghajari MF, Golpayegani MV, Bargrizan M, Ansari G, Shayeghi S. Sedative effect of oral midazolam/hydroxyzine versus chloral hydrate/hydroxyzine on 2-6 year-old uncooperative dental patients: a randomized clinical trial. J Dent (Tehran). 2014; 11(1):93-9. Guelmann M, Brackett R, Beavers N, Primosch RE. Effect of continuous versus interrupted administration of nitrous oxide-oxygen inhalation on behavior of anxious pediatric dental patients: a pilot study. J Clin Pediatr Dent. 2012;37(1):77-82. Guinsburg R, de Almeida PJ, de Araujo Peres C, Shinzato AR, Kopelman BI. Reliability of two behavioral tools to assess pain in preterm neonates. Sao Paulo Med L 2003;121:72-6. Hicks CL, von Baeyer CL, Spafford P, van Korlaar I, Goodenough B. The Faces Pain Scale - Revised: Toward a common metric in pediatric pain measurement. Pain. 2001; 93(2):173-183. Hill ML, Craig KD. Detecting deception in facial expressions of pain: accuracy and training. Clin J Pain. 2004;20(6):415-422. Houpt MI, Weiss NJ, Koenigsberg SR, Desjardins PJ. Comparison of chloral hydrate with and without promethazine in the sedation of young children.Pediatr Dent. 1985;7(1):41-6. Kunz M, Prkachin K, Lautenbacher S. The smile of pain. Pain. 2009;145(3):273-275. Larochette AC, Chambers CT, Craig KD. Genuine, suppressed and faked expressions of pain in children. Pain. 2006;126(1-3):64-71. Lourenço-Matharu L, Ashley PF, Furness S. Sedation of children undergoing dental treatment. Cochrane Database Syst Rev. 2012 Mar 14;3:CD003877. Mason KP, Green SM, Piacevoli Q; International Sedation Task Force. Adverse event reporting tool to standardize the reporting and tracking of adverse events during procedural sedation: a consensus document from the World SIVA International Sedation Task Force. Br J Anaesth. 2012;108(1):13-20. Merkel SI, Voepel-Lewis T, Malviya S. Pain assessment in infants and young children: the FLACC scale. Am J Nurs. 2002;102: 55-8. Millar K, Asbury AJ, Bowman AW, Hosey MT, Martin K, Musiello T, Welbury RR. A randomised placebo-controlled trial of the effects of midazolam premedication on children's postoperative cognition. Anaesthesia. 2007;62(9):923-30. Mittal N, Goyal A, Gauba K, Kapur A, Jain K. A double blind randomized trial of ketofol versus propofol for endodontic treatment of anxious pediatric patients. J ClinPediatrDent. 2013;37(4):415-20. Moreira TA, Costa PS, Costa LR, Jesus-França CM, Antunes DE, Gomes HS, Neto OA. Combined oral midazolam-ketamine better than midazolam alone for sedation of young children: a randomized controlled trial. Int J PaediatrDent. 2013;23(3):207-15. Oliveira FCM. Compreendendo a fobia em odontopediatria por meio das intervenções com o Procedimento de Desenho-Estória. 2008. 227 f. Dissertação (Mestrado) - Curso de Piscologia, Departamento de Instituto de Psicologia, Universidade de São Paulo, São Paulo, 2008. Pandey RK, Bahetwar SK, Saksena AK, Chandra G. A comparative evaluation of drops versus atomized administration of intranasal ketamine for the procedural sedation of young uncooperative pediatric dental patients: a prospective crossover trial. J ClinPediatr Dent. 2011;36(1):79-84. Shabbir A, Bhat SS, SundeepHegde K, Salman M. Comparison of oral midazolam and triclofos in conscious sedation of uncooperative children. J ClinPediatrDent. 2011;36(2):189-96. Somri M, Parisinos CA, Kharouba J, Cherni N, Smidt A, Abu Ras Z, Darawshi G, Gaitini LA. Optimising the dose of oral midazolam sedation for dental procedures in children: a prospective, randomised, and controlled study. Int J Paediatr Dent. 2012;22(4):271-9. Toomarian L, Salem K, Ansari G. Assessing the sedative effect of oral vs submucosalmeperidine in pediatric dental patients. Dent Res J (Isfahan). 2013;10(2):173-9. Tyagi P, Tyagi S, Jain A. Sedative effects of oral midazolam, intravenous midazolam and oral diazepam in the dental treatment of children. J ClinPediatrDent. 2013;37(3):301-5. Silva FC, Thuler LCS. Tradução e adaptação transcultural de duas escalas para avaliação da dor em crianças e adolescentes. J Pediatr (Rio J) 2008, 84,(4):344-9. Stewart SH, Buffett-Jerrott SE, Finley GA, Wright KD,

Valois Gomez T. Effects of midazolam on explicit vs implicit memory in a pediatric surgery setting. Psychopharmacology (Berl). 2006 Nov;188(4):489-97. Tardivo LSLC. O procedimento de desenhos-estórias (D-E) e seus derivados: fundamentação teórica, aplicações em clínica e pesquisas. In: Villemor-Amaral AE; Werlang BSG. Atualizações em Métodos Projetivos para Avaliação Psicológica. São Paulo: Casa do Psicólogo, 2011. p. 287-304. Trinca W. Formas de investigação clínica em psicologia: procedimento de desenhos-estórias: procedimento de desenhos de família com estórias. Vetor, 1997. Martins-Júnior PA, Ramos-Jorge J, Paiva SM, Marques LS, Ramos-Jorge ML. Validations of the Brazilian version of the Early Childhood Oral Health Impact Scale (ECOHIS). Cad Saude Publica 2012; 28 (2):367-74.

Upload de Documentos

Arquivo Anexos:

Tipo	Arquivo
Folha de Rosto	NASO - folha de rosto CONEP.pdf
Outros	Apendice F_eventos adversos.doc
Outros	PendenciaE1_carta_encaminhamento.pdf
Outros	PendenciaE1_Emenda_corrigida_15_03_2017.pdf
Outros	PendenciaE1_Relatorio_Parcial.pdf
Projeto Detalhado / Brochura Investigador	projeto NASO_v 15_09_14.pdf
Outros	Apendice C_justificativa_dispensa_assentimento.docx
TCLE / Termos de Assentimento / Justificativa de Ausência	Apendice B3.TCLE_oped.docx
Comprovante de Recepção	PB_COMPROVANTE_RECEPCAO_872941.pdf
Declaração de Pesquisadores	NOVO_compromisso_pesqusiadores.pdf
Outros	Apendice D_recomendacoes pre e pos.docx
Outros	E2_Relatorio_Parcial.pdf
Outros	Anexo A_Declaracao_SERUPE.pdf
Outros	ApEndice E_fichas de avaliacao.docx
TCLE / Termos de Assentimento / Justificativa de Ausência	PendenciaE1_TCLEs_15_03_2017.doc
Recurso Anexado pelo Pesquisador	CNPq_Universal_2014_NASO_termo_aceite.pdf

Finalizar

Manter sigilo da integra do projeto de pesquisa: Não

Justificativa da Emenda:

Prezados Coordenadores do CEP/UFG: Solicito, na presente emenda E2, a extensão do prazo para execução do projeto para 31/12/2019 (itens do cronograma análise dos dados, pesquisa bibliográfica e produção dos documentos científicos relacionados abaixo, com base no que se segue: Necessita-se de maior prazo com a finalidade de explorar análises associadas aos objetivos já estabelecidos no projeto, escrever e submeter artigos, relatórios e dissertações/teses, tendo em vista que este projeto se trata de ensaio clínico com uma variedade de objetivos específicos, os quais exigem dedicação maior por alunos de iniciação científica e pós-graduação estrito sensu. Especificamente, os objetivos específicos que ainda necessitam de análises e redação científica mais aprofundadas são: • Aceitação da administração do sedativo: a ocorrência de dor relacionada à administração do sedativo está em fase de início de análise dos vídeos, sob a responsabilidade de Anelise Daher Vaz Castro. • Comportamento das crianças durante o procedimento odontológico: especificamente comparando o comportamento durante o exame clínico e o procedimento restaurador, objeto da dissertação de mestrado de Mônica Alves Moterane, a ser concluída em fevereiro/2019. • Memória do procedimento: os dados já foram coletados e estão em fase de análise para posterior relatório científico, que se constituirá em parte da tese de doutorado de Karolline Alves Viana, a concluir possivelmente no final de 2019. • Nível de cortisol salivar: a saliva dos participantes foi coletada, mas falta analisar a saliva das crianças e associá-la a outras variáveis. • Analisar a percepção da criança acerca do tratamento odontológico sob sedação: os dados já foram coletados e estão em fase de análise para posterior relatório científico, que se constituirá em parte da tese de doutorado de Karolline Alves Viana, a concluir possivelmente no final de 2019. • Realizar análise de custo-efetividade dos diferentes regimes sedativos. Está em desenvolvimento, sob a responsabilidade de Liliani Aires Cândido Vieira. Anexamos, nesta solicitação, relatório parcial das atividades executadas até o momento. Atenciosamente, Profa. Luciane R. R. S. Costa
