# Supplementary material for: Randomized clinical trial on the efficacy of intranasal or oral ketamine-midazolam combinations compared to oral midazolam for outpatient pediatric sedation
Source: PLoS One. 2019 Mar 11;14(3):e0213074. doi: 10.1371/journal.pone.0213074 (PMC6411109; doi:10.1371/journal.pone.0213074)
Supplement: S2 Protocol — (PDF) [file pone.0213074.s004.pdf]

Research project:
Efficacy, safety and cost-effectiveness of intranasal sedation with ketamine and midazolam in pediatric dentistry: randomized clinical trial

Preliminary information

Principal Investigator

CPF/Document: 510.126.341-91	Name: Luciane Ribeiro de Rezende Sucasas da Costa
Telephone: 6232096325	E-mail: lsucasas@ufg.br

Proponent Institution

CNPJ:	Institution's name: Dental School, Universidade Federal de Goias

Is this amendment submission exclusive to your Coordinating Center?

The amendment is exclusive to your Coordinating Center, so any changes made to your project by virtue of the amendment will NOT be replicated at the Linked Participating Centers and the Ethics Committees of the Coparticipant Institutions upon their approval.

Is it an international study? No

Research Team

CPF/Document	Name
284.341.228-55	Aline Carvalho Batista
042.947.331-10	Kárita Cristina Silva
480.068.231-20	Geovanna de Castro Morais Machado
180.922.628-70	Liliani Aires CAndido Vieira
019.437.701-66	JOJI SADO FILHO
024.142.381-35	Karolline Alves Viana
025.787.211-65	Anna Alice Anabuki
081.855.646-39	Heloisa de Sousa Gomes
802.184.901-00	Anelise Daher Vaz Castro
383.187.171-04	Paulo Sérgio Sucasas da Costa
084.368.856-43	Patrícia Corrêa de Faria
252.503.291-87	Mara Rúbia de Camargo Alves Orsini
036.674.591-30	Mônica Maia Moterane

Study Area

Great Areas of Knowledge (CNPq)

- Great Area 4. Health Science

Main Purpose of the Study (WHO) Clinical

-

Public Research Title: Efficacy, safety and cost-effectiveness of intranasal sedation with ketamine and midazolam in pediatric dentistry: randomized clinical trial

Expansion of the Public Acronym: Nasal administration of sedation in dentistry

Public Contact

CPF/Document	Name	Telephone	E-mail
510.126.341-91	Luciane Ribeiro de Rezende Sucasas da Costa	6232096325	lsucasas@ufg.br

Scientific Contact: Luciane Ribeiro de Rezende Sucasas da Costa

Study Design / Funding

Study Design: Intervention/Experimental

Health conditions or problems

Health condition or Problem
Dental anxiety
Behavioral dental problems
Dental care for children
Early childhood caries

General Descriptors for Health Conditions

ICD-10 International Classification of Diseases

ICD Code	ICD Description
R52.0	Acute pain
F41.1	Generalized anxiety disorder

MESH: Medical Subject Headings

MESH Code	MESH Description
E03.250	Conscious sedation
E06.170.152	Dental care for children

Specific Descriptors for Health Conditions

ICD-10 International Classification of Diseases

ICD Code	ICD Description
K02	Dental caries

MESH: Medical Subject Headings

MESH Code	MESH Descriptor
F01.470.132.300	Dental anxiety

Type of intervention: Comparator

Nature of Intervention

- Drug / Medicine / Vaccine

Intervention Descriptors

Intervention Descriptor

Interventions
Intranasal administration of ketamine and midazolam
Oral administration of ketamine and midazolam
Oral administration of midazolam
Midazolam

ICD list

ICD Code	ICD Descriptor
F41.1	Generalized anxiety disorder

MESH List

MESH Code	MESH Descriptor
E03.250	Conscious sedation

Phase

- Phase 4

Will there be use of placebo or the existence of groups that will not be submitted in any intervention?

There will be use of placebo only to compare routes of intranasal and oral administration, however all children will receive sedative for dental treatment. That is, when the child receives sedative intranasally, s/he will receive placebo by mouth, and vice versa.

Design:

Randomized clinical trial with masking and parallel design

Financial Support

CNPJ	Name	E-mail	Telephone	Type
				Own financing
01.263.896/0004-07	MINISTRY OF SCIENCE, TECHNOLOGY AND INNOVATION		2121232703	Main Institutional

Keywords

Keyword
Midazolam
Conscious sedation
Pediatric dentistry
Ketamine

Study

Detail

Summary:

About 10% of children may exhibit behavioral problems during dental treatment, but the evidence for sedation protocols that benefit pediatric dentistry care is still poor. In this randomized, masked and controlled, parallel-designed clinical trial, the efficacy, safety and cost-effectiveness of intranasal sedation with ketamine / midazolam in healthy 2- to 6-year-old children is sought. Children will be selected from those with dental caries that do not allow care in two visits, and require at least two restorations under local anesthesia and absolute isolation. A preliminary sample of 84 children equally distributed in three groups: A) ketamine (4.0 mg / kg, maximum 100 mg) + midazolam (0.2 mg / kg, maximum 5 mg) intranasally; B) ketamine (4.0 mg / kg, maximum 100 mg) + midazolam (0.5 mg / kg, maximum 20 mg) orally; C) midazolam (1.0 mg / kg, maximum 20 mg) orally. The hypothesis is that ketamine / midazolam, administered intranasally, is an effective, safe and cost-effective sedative regimen for use in pediatric dentistry in the public service. The primary endpoint is the behavior of the child assessed minute by minute, according to the Houpt scale, in the files of the sedation session films. The secondary outcomes are: acceptance of the sedative administration, memory of the procedure and level of salivary cortisol, within each intervention group. In addition, we will evaluate: pain occurrence and association of changes in facial expressions with potentially painful stimuli in sedated children during dental care; perception of the companions (n = 84) and the dentist (n = 6) on sedation, through self-report and stress measured by salivary cortisol; perception of the child about dental treatment under sedation, through a projective test; adverse events occurring during and after administration of sedatives; cost-effectiveness of different sedative protocols. The proposed experimental design aims to avoid systematic and random errors, to contribute with a higher level of evidence in future systematic reviews. The outcomes of this study have a potential impact on public and private health practice, and may serve as guidance for conducts in institutional guidelines linked to the Unified Health System and opinion-forming entities.

Introduction:

2.1. Reserch question: What is the efficacy, safety and cost of intranasal sedation with ketamine / midazolam for children with behavioral problems during dental treatment?
2.2 Relevance: Behavioral problems during the dental treatment of children and adolescents afflict about 9% of the patients who are treated with oral ketamine / midazolam, of the world's population. Although they decrease with age and are more observed in girls, these behavioral problems are associated with general fears, externalizing and internalizing behavioral problems (Klingberg et al.
2007). The non-cooperative approach to the dentist's chair is traditionally done by non-pharmacological (physical restraint) or pharmacological methods (sedation or general anesthesia), associated with communicative techniques that encourage the child to cope with the situation. Decision-making to choose the type of approach depends on: dental need, child developmental stage, dentist training, physical and human resources available and choice of parents or caregivers. The physical restraint, modernly called protective stabilization, may be indicated in situations of need for immediate conduct and preferably associated with a sedative, to avoid psychological trauma to the child (American Academy of Pediatric Dentistry "AAPD", 2013-2014). The contraindication to the routine use of physical restraint is due to the fact that this constitutes a form of violence against the child. One of the recommendations of the National Plan for Early Childhood (2010, page 68) is "Recognition of physical and humiliating punishment as forms of violence against children and is therefore a violation of their fundamental rights with an impact on healthy child development. "Sedation aims to calm the patient, promote sleep and / or increase the pain threshold. Still, it causes depression of consciousness at different levels - minimal, moderate, profound. Most sedatives have the potential to cause amnesia, which favors the impact on the child's reactions after a treatment in which the sedative was not effective. Moderate sedation has been best indicated for pre-cooperative or mild anxiety children, as long as dental need does not require a large number of sessions to complete treatment. In this sense, the success rate of sedation in pediatric dentistry is variable, highly dependent on sedation (Table 1). Since it is performed under a well-established protocol, the occurrence of adverse events associated with sedation is low. General anesthesia is the preferred method for very young children, unable to co-operate even with sedation. present an extensive need for dental treatment (AAPD 2013-2014). However, due to the greater demands for general anesthesia, it becomes a more costly procedure from the point of view of human, structural and financial resources, which justifies the search for effective alternatives for the behavioral management of children with behavioral problems during dental treatment Dental caries is still the major cause of need for dental treatment in children and, despite having a declining prevalence in some countries, it is a disease that still affects thousands of people around the world. In Brazil, despite the government's significant investments and the decline in prevalence rates, 53.4% of Brazilian children have caries in the deciduous dentition. At the age of five, a Brazilian child has, on average, 2.43 teeth with caries experience, with predominance of the carious component, which accounts for more than 80% of the index (Brazil, 2010).
2.3 Rationale: Databases of scientific articles show that many researchers and clinicians are concerned with identifying the sedative protocol that promotes the highest possible comfort in pediatric dentistry, with a lower risk. According to the PubMed database (dental, sedation, child), there are 785 articles published from 1965 to 06/07/2014 on this subject. However, when this search is restricted to randomized controlled clinical trials (RCTs), the number drops to 117 articles. Lourenço-Matharu et al. conducted a systematic review of the Cochrane database published in 2012 to evaluate the effectiveness of agents and doses in moderate sedation for behavioral management in pediatric dentistry. They identified 36 studies published until 04/11/2011, which contained biases.

They concluded that there is weak evidence that oral midazolam is an effective sedative agent for children undergoing dental treatment, and very weak evidence in favor of nitrous oxide. The authors also found that it is necessary to conduct clinical trials with adequate design to evaluate other potential sedation agents, and to evaluate experimental regimens compared to oral midazolam or inhaled sedation with nitrous oxide. Since then, 10 RCTs on sedation in pediatric dentistry were published, investigating different protocols for different age groups (Table 1). However, most of them still commit methodological inadequacies such as: cross-delineation; evaluations of outcomes by untrained / calibrated examiners; lack of analysis of pain, stress, memory, cost of the procedure under sedation, and other aspects of the perception of the participants (child / companion) on the success of sedation.Therefore, it is justified the re-reading of sedation in pediatric dentistry through RCT that tries to evaluate different indicators related to the success of sedation. Our research group has obtained good results with the ketamine / midazolam combination (Moreira et al., 2013), resulting from a history of studies with other drugs. As the intranasal use of drugs seems to result in better results than the oral one, the focus of this research will be to investigate the association of ketamine / midazolam with this route.

Hypothesis:

The hypothesis is that ketamine / midazolam, administered intranasally, is an effective, safe and cost-effective sedative regimen for use in pediatric dentistry in the public service.

Primary Aim:

To investigate sedative regimens with ketamine and midazolam that benefit the dental treatment of preschool children.

Secondary Aim:

To compare the efficacy of intranasal administration of ketamine / midazolam to oral administration of ketamine / midazolam and midazolam in children 2 to 6 years of age, using the following indicators:

- Acceptance of sedative administration.
- Behavior of the children during the dental procedure.
- Procedure memory.
- Salivary cortisol level.

To evaluate the occurrence of pain and the association of changes in facial expressions with potentially painful stimuli in sedated children during dental care.

To seek the perception of the caregivers and the dentist on the sedation, through self-report and stress measured by salivary cortisol.

To analyze the child's perception about dental treatment under sedation.

To detect adverse events occurring during and after the administration of sedatives.

To carry out a cost-effectiveness analysis of the different sedative regimens.

To verify the impact of dental caries and associated factors, as well as dental sedation, on the perception of the companions on the oral health related quality of life (OHRQOL) of non-cooperative preschoolers with dental treatment.

Proposed Methodology:

STUDY TIPE: Randomized, masked and controlled clinical trial of a parallel design. In this amendment, considering the specific objective mentioned in item 1, it is observed the necessity of a prospective longitudinal design. For this, the children included in the clinical trial and their caregivers or companions will undergo periodic evaluations to verify changes in the Oral Health Related Quality of Life (OHRQOL). The evaluations will be carried out in three moments, the first one (baseline-T0) before the treatment and the other in the period of two weeks and three months after the conclusion of the dental treatment. From the baseline scores, the sample will be divided into two groups, through cluster analysis, according to a higher or lower score observed in the B-ECOHIS instrument. This analysis will be performed after obtaining the initial data of the entire sample.
STUDY LOCATION: This study will be carried out at the Dental School of the Federal University of Goiás (FO / UFG), in the extension project "Nucleus of Studies in Dental Sedation" (NESO). NESO that has the necessary infrastructure to carry out dental treatment under sedation, namely: complete dental equipment; heart rate monitors, oxygen saturation (pulse oximeter), carbon dioxide (capnography), and blood pressure; sedatives and respective antagonistic drugs; material for administering drugs by oral and intravenous route; material for orotracheal intubation; oxygen cylinder; anesthesia cart; emergency drug kit (examples: adrenaline, hydrocortisone, diazepam, glucose, atropine, etc.). In addition, NESO integrates a multidisciplinary team trained and qualified to act in dental sedation: dental surgeon, pediatrician, anesthesiologist, pediatric dentist, psychologist, bucomaxillofacial surgeon. In terms of public health, NESO is unique in the country. Historically, NESO has made it possible to carry out research at the undergraduate and postgraduate level that has reached publications of impact in the area. In this way, NESO provides a safe environment for conducting research aimed at sedating children.
ETHICAL ASPECTS: This research will follow the precepts of Resolution CNS / MS 466/2012. Once approved by the Research Ethics Committee, the project will be recorded in a clinical trial database and data collection will commence. Other considerations are described in the project.
PARTICIPANTS: Study population: children from 2 to 6 years old with dental caries, referred with a history of non-cooperation with dental care. Sampling: non-probabilistic - convenience sample of children referred from public services in Goiânia-GO and
(Appendix A) with succinct information about the research will be distributed at health clinics and dental specialty centers, directing health professionals to refer potential research participants to NESO.
INTERVENTIONS: This clinical trial will compare the effect of three types of intervention (Table 2). In this way, all the children will receive a sedative regime, either intranasal or oral. Group A, experimental, still lacks results in the literature and justifies the present investigation. Groups B and C are sedative regimens that have positive evaluations in the literature and will serve to compare the experimental regimen. Only the doctor will know the group to which the child was drawn because of the need to take immediate action in case of an adverse event. Thus, the child / companion, dentist operator and auxiliary and other observers will be masked for the intervention group.
DATA COLLECTION PROCEDURES: Through dental consultations, application of questionnaires and interviews.

Inclusion Criteria:

Children whose physical condition is categorized as ASA I (healthy) or II (mild and controlled systemic disease - persistent asthma, for example); children with a low risk of airway obstruction (Malampati less than 2 and / or tonsil hypertrophy occupying less than 50% of the oropharynx); absence of medical history of neurological or cognitive alterations; absence of facial deformities; birth to term; children who do not use medications that may compromise cognitive functions; children with at least two teeth with caries without pulp involvement, requiring dental restoration under local anesthesia and absolute isolation.

Exclusion Criteria:

Children with positive or definitely positive behavior (Frankl et al., 1962) in a dental treatment session performed by the trained and calibrated team.

Risks:

Prior to the data collection, the legal guardians of the children or companions will be clarified by the researcher about the research objectives, risks and benefits. The risks and discomforts expected of the research participants (child) are those inherent to dental treatment under sedation. Adverse events related to sedation in NESO are infrequent and of minor severity (Costa et al., 2012). Adverse events may occur during the procedure or within the first 4 hours after the administration of the sedatives. These adverse events may be of minimal risk, low risk or high risk / sentinel (Table 2). In case they occur, the procedures established in the consensus of the International Sedation Task Force (ISTF) of the World Society of Intravenous Anesthesia (World SIVA) will be adopted. The first care will be performed at NESO and, if necessary, a member of the team will accompany the patient to the emergency room of the UFG Hospital, according to the signed agreement (Appendix D). Although adverse home-based adverse events are rare, those responsible will be verbally and in writing advised about the postoperative recommendations (Appendix E), emphasizing the need to contact the team in case of doubt. If necessary, the staff will go to the patient's home to evaluate it or, in more serious cases, will activate the Emergency Service (192) and follow up all patient care. If adverse events occur at any point in the study, the need to adjust or discontinue the study will be assessed. However, the resources and protocols adopted in NESO aim to minimize the occurrence of major adverse events because they are based on scientific evidence and apply the ethical principles of beneficence, non-maleficence and otherness. The risks for companion and dentist, who will have their saliva collected and will respond to two questions in a questionnaire, will be a breach of privacy in the collection of information or confidentiality in the dissemination of results, but the research team will take precautions to prevent this: password-encrypt access to databases, and not the name of the participant in the column of identification of the cases in the database. For the specific purpose that was added in this amendment, the risks of the study are related to the possible embarrassment of the responsible ones or companions in answering the questionnaire. The risk of embarrassment will be minimized by providing the responsible or accompanying persons with an environment conducive to the application of the questionnaire, complete freedom of reply and questioning, as well as guaranteeing the confidentiality of their name and image.

Benefits:

The benefits will be direct and indirect. The completion of dental treatment and subsequent monitoring of oral condition will directly benefit the child. The scientific community will benefit from the evidence produced in the study that should be applied to populations similar to the sample evaluated. The results of this research have the potential to benefit other children who resemble the conditions of this sample, to the extent that an effective sedative regimen is identified. The results of this study will be published, whether they are favorable or not, and will contribute to the scientific knowledge currently available. The caregivers or caregivers may choose to withdraw their participation in the study without any harm to their relationship and that of the child with the institution and the research team. This information, as well as the description of the research, are contained in the Informed Consent Form, which will be signed by the parents or guardians in case of agreement on their participation and that of the child in the study (APPENDICES A, B, C).

Data Analysis Methodology:

SAMPLE SIZE: The sample size was calculated based on the primary variable, that is, the behavior of children during sedation. In the study by Moreira et al. (Median 8.6, standard deviation 4.1), midazolam (14.0; 3.8), both administered orally, were measured in the midazolam / ketamine groups and no sedative (12.5, 5.2). Based on these values, it was calculated in 23 cases per group, comparing the midazolam / ketamine groups and no sedative, to obtain a test power of 80% at the 5% level. Thinking of a loss of 20%, a preliminary sample of 28 children per group is estimated. However, as no parallel-design clinical trial has been found to make the same comparisons of this design, this calculation will be confirmed by a pilot study to be performed with the first randomized block of cases.

DATA ANALYSIS: Descriptive and bivariate analyzes will be performed in Prisma GraphPad and IBM SPSS statistical software, considering the significance level of 5%. After confirming the distribution of continuous variables (normal / non-normal), the three groups will be compared according to the different outcome variables observed at the first intervention visit.

Primary Outcome:

The primary outcome measure will be the child's behavior during sedation treatment, but there will be secondary outcome measures that will contribute to a more comprehensive understanding of the success of sedation.

Secondary Outcomes:

Acceptance of sedative administration. Pain during treatment. Memory. Companion and dentist perception about sedation. Perception of the child about sedation. Adverse events.

Sample Size in Brazil: 174

Recruitment Countries

Country of Origin of the Study	Country	N° of research participants
Yes	BRAZIL	174

Other Information

Will there be use of secondary sources of data (medical records, demographics, etc.)?

No

Please state the number of individuals approached personally, recruited, or who will undergo any type of intervention at this research center:

174

Groups in which the research participants will be divided in this center:

Group ID	Nº of Subjects	Interventions to be carried out
Mothers/caregivers	84	Respond to quality of life questionnaires; saliva collected for the evaluation of salivary cortisol
Group A	28	Children - intranasal sedation (ketamine / midazolam) + oral placebo; companions - will respond to questionnaire and saliva collection
Group B	28	Children - placebo intranasal sedation + oral sedation (midazolam / ketamine); companions - will respond to questionnaire and saliva collection
Group C	28	Children - placebo intranasal sedation + oral sedation (midazolam); companions - will respond to questionnaire and saliva collection
Dentists	6	They will answer the questionnaire and have saliva collection

Is the Study Multicentric in Brazil??

No

Do you propose exemption from the Informed Consent?

Yes

Justification:

Researchers ask for exemption from free and informed consent for this stage of the research. The dispensation is necessary because the children who participate in the study have non-cooperative behavior in dental care and this could result in a large proportion of refusal to participate. In addition, another limitation of the application of the assent term is the difficulty of understanding the objectives of the research by children who are still in the stage of cognitive development. The researchers, recognizing that this research will involve vulnerable human beings, undertake to follow the ethical precept of autonomy of the child, respecting their willingness to participate in research and ensure their privacy. The physical, psychological and moral integrity of these participants will also be guaranteed, as established in the Statute of the Child and Adolescent.

Will there be sample retention for bank storage?

No

Project Timeline

Step Identification	Start (DD/MM/YYYY)	End (DD/MM/YYYY)
Registration in a clinical trial database	17/11/2014	21/11/2014
Monitoring (post-treatment evaluations completion)	17/04/2017	31/12/2017
Bibliographic research	01/04/2018	31/12/2019
Analysis of saliva samples	02/02/2015	31/03/2017
Conduct of dental consultations	01/04/2017	30/11/2017
Team training and calibration	06/10/2014	14/11/2014
Data collection	01/12/2014	16/12/2016
Recruiting	24/11/2014	31/08/2016
Production of scientific documents: abstracts, dissertations, theses and reports	01/04/2018	31/12/2019
Data analysis	01/04/2018	31/12/2019
Watching Videos	02/02/2015	31/03/2017
Carry out interventions	01/12/2014	16/12/2016

Financial Budgeting

Budget Identification	Type	Value, Brazilian Real (R$)
External Hard Drives for Data Storage	Capital	R$ 1.000,00
Total, R$		R$ 1.000,00

Tablet	Capital	R$ 950,00
Drugs related to sedation (including placebo)	Cost	R$ 2.000,00
Portable Digital Video Cameras (2 pcs)	Capital	R$ 3.000,00
Digital audio recorder	Capital	R$ 250,00
Transportation reimbursement for caregivers	Cost	R$ 1.500,00
Office Supplies	Cost	R$ 2.000,00
Total em R$		R$ 10.700,00

Bibliography:

American Academy on Pediatric Dentistry Clinical Affairs Committee-Behavior Management Subcommittee. Guideline on behavior guidance for the pediatric dental patient. Pediatr Dent. 2013-2014;35(6):176-87. American Academy of Pediatrics; American Academy on Pediatric Dentistry. Guideline for monitoring and management of pediatric patients during and after sedation for diagnostic and therapeutic procedures. Pediatr Dent. 2013-2014;35(6):205-21. Ashraf AB, Lucey S, Cohn JF, Chen T, Ambadar Z, Prkachin KM, Solomon PE. The painful face – pain expression recognition using active appearance models. Image Vis Comput. 2009;27(12):1788-1796. Bahetwar SK, Pandey RK, Saksena AK, Chandra G. A comparative evaluation of intranasal midazolam, ketamine and their combination for sedation of young uncooperative pediatric dental patients: a triple blind randomized crossover trial. J ClinPediatr Dent. 2011;35(4):415-20. Bhatnagar S, Das U M, Bhatnagar G. Comparison of oral midazolam with oral tramadol, triclofos and zolpidem in the sedation of pediatric dental patients: An in vivo study. J Indian SocPedodPrev Dent 2012;30:109-14. Birnie KA, Noel M, Chambers CT, von Bayer CL, Fernandez CV. The cold pressor task: is it an ethically acceptable pain research method in children? J PediatrPsychol 2011;13:1071-81. Birnie KA, Petter M, Boerner KE, Noel M, Chambers CT. Contemporary use of the cold pressor task in pediatric pain research: a systematic review of methods. J Pain 2012;13:817-26. Boerner KE, Chambers CT, Craig KD, Pillai Riddell RR, Parker JA. Caregiver accuracy in detecting deception in facial expressions of pain in children. Pain. 2013;154(4):525-533. Brasil. Ministério da Saúde, Departamento de Atenção Básica (DAB). Projeto SBBrasil 2010: Pesquisa Nacional de Saúde Bucal: resultados principais. Brasília: Ministério da Saúde, 2011. Brasil. Ministério da Saúde. Secretaria-Executiva. Área de Economia da Saúde e Desenvolvimento. Avaliação de tecnologias em saúde: ferramentas para a gestão do SUS / Ministério da Saúde, Secretaria-Executiva, Área de Economia da Saúde e Desenvolvimento. – Brasília: Ministério da Saúde, 2009. Buffett-Jerrott SE, Stewart SH, Finley GA, Loughlan HL. Effects of benzodiazepines on explicit memory in a paediatric surgery setting. Psychopharmacology (Berl). 2003 Aug;168(4):377-86. Capovilla FC, Negrão V, Damázio M, Roberto MR, Marins KC, Sousa-Sousa CC, Lima-Sousa A, Botelho F. Pictografia evocadora de fala: CD2: BFI-Livres: 3100 figuras com normatização de nomeação do maternal ao ensino superior, e do grau de familiaridade do nome no ensino fundamental. 1. ed. São Paulo, SP: Memnon, 2011. v. 1. Chambers CT, Cassidy KL, McGrath PJ, Gilbert CA, Craig KD. Child Facial Coding System Manual. Halifax, Nova Scotia: Dalhousie University and University of British Columbia, 1996. Chang J, Versloot J, Fashler SR, McCrystal KN, Craig KD. Pain assessment in children: validity of facial expression items in observational pain sclaes. Clin J Pain. 2014 [Epub ahead of print]. Chopra R, Mittal M, Bansal K, Chaudhuri P. Buccal midazolam spray as an alternative to intranasal route for conscious sedation in pediatric dentistry. J ClinPediatrDent. 2013;38(2):171-3. Costa LR, Costa PS, Brasileiro SV, Bendo CB, Viegas CM, Paiva SM. Post-discharge adverse events following pediatric sedation with high doses of oral medication. J Pediatr. 2012 May;160(5):807-13. Didonet V. Plano Nacional pela Primeira Infância. Brasília: Rede Nacional Primeira Infância, 2010. Disponível em: http://primeirainfancia.org.br/wp-content/uploads/PPNI-resumido.pdf>. Acesso em 07 jun. 2014. Cunha JA. Desenvolvimentos do Procedimento de Desenhos-Estórias (D-E). In: CUNHA, Jurema Alcides. Psicodiagnóstico-V. 5. ed. São Paulo: Artmed, 2000. Cap. 29, p. 248-249. Drummond MF, O'Brien B, Stoddart GL, Torrance GW: Methods for the economic evaluation of health care programmes. 3rd ed. New York: Oxford University Press; 2005. Frankl S, Shiere F, Fogels H. Should the parent remain with the child in the dental operatory. J Dent Child. 1962;29:150-163. Freitas NK. CAT e sua interpretação dinâmica. In: Cunha JA. Psicodiagnóstico-V. 5. ed. São Paulo: Artmed, 2000. Cap. 27, p. 416-420. Ghajari MF, Golpayegani MV, Bargrizan M, Ansari G, Shayeghi S. Sedative effect of oral midazolam/hydroxyzine versus chloral hydrate/hydroxyzine on 2-6 year-old uncooperative dental patients: a randomized clinical trial. J Dent (Tehran). 2014; 11(1):93-9. Guelmann M, Brackett R, Beavers N, Primosch RE. Effect of continuous versus interrupted administration of nitrous oxide-oxygen inhalation on behavior of anxious pediatric dental patients: a pilot study. J Clin Pediatr Dent. 2012;37(1):77-82. Guinsburg R, de Almeida PJ, de Araujo Peres C, Shinzato AR, Kopelman BI. Reliability of two behavioral tools to assess pain in preterm neonates. Sao Paulo Med L 2003;121:72-6. Hicks CL, von Baeyer CL, Spafford P, van Korlaar I, Goodenough B. The Faces Pain Scale - Revised: Toward a common metric in pediatric pain measurement. Pain. 2001; 93(2):173-183. Hill ML, Craig KD. Detecting deception in facial expressions of pain: accuracy and training. Clin J Pain. 2004;20(6):415-422. Houpt MI, Weiss NJ, Koenigsberg SR, Desjardins PJ. Comparison of chloral hydrate with and without promethazine in the sedation of young children.Pediatr Dent. 1985;7(1):41-6. Kunz M, Prkachin K, Lautenbacher S. The smile of pain. Pain. 2009;145(3):273-275. Larochette AC, Chambers CT, Craig KD. Genuine, suppressed and faked expressions of pain in children. Pain. 2006;126(1-3):64-71. Lourenço-Matharu L, Ashley PF, Furness S. Sedation of children undergoing dental treatment. Cochrane Database Syst Rev. 2012 Mar 14;3:CD003877. Mason KP, Green SM, Piacevoli Q; International Sedation Task Force. Adverse event reporting tool to standardize the reporting and tracking of adverse events during procedural sedation: a consensus document from the World SIVA International Sedation Task Force. Br J Anaesth. 2012;108(1):13-20. Merkel SI, Voepel-Lewis T, Malviya S. Pain assessment in infants and young children: the FLACC scale. Am J Nurs. 2002;102: 55-8. Millar K, Asbury AJ, Bowman AW, Hosey MT, Martin K, Musiello T, Welbury RR. A randomised placebo-controlled trial of the effects of midazolam premedication on children's postoperative cognition. Anaesthesia. 2007;62(9):923-30. Mittal N, Goyal A, Gauba K, Kapur A, Jain K. A double blind randomized trial of ketofol versus propofol for endodontic treatment of anxious pediatric patients. J ClinPediatrDent. 2013;37(4):415-20. Moreira TA, Costa PS, Costa LR, Jesus-França CM, Antunes DE, Gomes HS, Neto OA. Combined oral midazolam-ketamine better than midazolam alone for sedation of young children: a randomized controlled trial. Int J PaediatrDent. 2013;23(3):207-15. Oliveira FCM. Compreendendo a fobia em odontopediatria por meio das intervenções com o Procedimento de Desenho-Estória. 2008. 227 f. Dissertação (Mestrado) - Curso de Piscologia, Departamento de Instituto de Psicologia, Universidade de São Paulo, São Paulo, 2008. Pandey RK, Bahetwar SK, Saksena AK, Chandra G. A comparative evaluation of drops versus atomized administration of intranasal ketamine for the procedural sedation of young uncooperative pediatric dental patients: a prospective crossover trial. J ClinPediatr Dent. 2011;36(1):79-84. Shabbir A, Bhat SS, SundeepHegde K, Salman M. Comparison of oral midazolam and triclofos in conscious sedation of uncooperative children. J ClinPediatrDent. 2011;36(2):189-96. Somri M, Parisinos CA, Kharouba J, Cherni N, Smidt A, Abu Ras Z, Darawshi G, Gaitini LA. Optimising the dose of oral midazolam sedation for dental procedures in children: a prospective, randomised, and controlled study. Int J Paediatr Dent. 2012;22(4):271-9. Toomarian L, Salem K, Ansari G. Assessing the sedative effect of oral vs submucosalmeperidine in pediatric dental patients. Dent Res J (Isfahan). 2013;10(2):173-9. Tyagi P, Tyagi S, Jain A. Sedative effects of oral midazolam, intravenous midazolam and oral diazepam in the dental treatment of children. J ClinPediatrDent. 2013;37(3):301-5. Silva FC, Thuler LCS. Tradução e adaptação transcultural de duas escalas para avaliação da dor em crianças e adolescentes. J Pediatr (Rio J) 2008, 84,(4):344-9. Stewart SH, Buffett-Jerrott SE, Finley GA, Wright KD,

Valois Gomez T. Effects of midazolam on explicit vs implicit memory in a pediatric surgery setting. Psychopharmacology (Berl). 2006 Nov;188(4):489-97. Tardivo LSLC. O procedimento de desenhos-estórias (D-E) e seus derivados: fundamentação teórica, aplicações em clínica e pesquisas. In: Villemor-Amaral AE; Werlang BSG. Atualizações em Métodos Projetivos para Avaliação Psicológica. São Paulo: Casa do Psicólogo, 2011. p. 287-304. Trinca W. Formas de investigação clínica em psicologia: procedimento de desenhos-estórias: procedimento de desenhos de família com estórias. Vetor, 1997. Martins-Júnior PA, Ramos-Jorge J, Paiva SM, Marques LS, Ramos-Jorge ML. Validations of the Brazilian version of the Early Childhood Oral Health Impact Scale (ECOHIS). Cad Saude Publica 2012; 28 (2):367-74.

Documents Upload

Attached Files:

Type	Name of the File
Cover Sheet	NASO - folha de rosto CONEP.pdf
Others	Apendice F_eventos adversos.doc
Others	PendenciaE1_carta_encaminhamento.pdf
Others	PendenciaE1_Emenda_corrigida_15_03_2017.pdf
Others	PendenciaE1_Relatorio_Parcial.pdf
Detailed Project / Brochure of the Investigator	projeto NASO_v 15_09_14.pdf
Others	Apendice C_justificativa_dispensa_assentimento.docx
Informed Consent / Terms of Assent / Absence Justification	Apendice B3.TCLE_oped.docx
Proof of Receipt	PB_COMPROVANTE_RECEPCAO_872941.pdf
Researchers' Statement	NOVO_compromisso_pesqusiadores.pdf
Others	Apendice D_recomendacoes pre e pos.docx
Others	E2_Relatorio_Parcial.pdf
Others	Anexo A_Declaracao_SERUPE.pdf
Others	ApEndice E_fichas de avaliacao.docx
Informed Consent / Terms of Assent / Absence Justification	PendenciaE1_TCLEs_15_03_2017.doc
Proof of Funding Attached by Researcher	CNPq_Universal_2014_NASO_termo_aceite.pdf

Finish

Maintain confidentiality of the research project: No

Justification of the Amendment:

Dear REB / UFG Coordinators: I am requesting, in this amendment E2, the extension of the deadline for the execution of the project for 12/31/2019 (schedule items, data analysis, bibliographic research and production of the scientific documents listed below, based on what as follows: It is necessary to extend the term in order to explore analyzes associated with the goals already established in the project, to write and submit articles, reports and dissertations / theses, considering that this project is a clinical trial with a variety of objectives Specifically, the specific objectives that still require further analysis and scientific writing are: • Acceptance of the administration of sedative: the occurrence of pain related to the administration of the sedative is in the beginning phase of analysis of the videos, under the responsibility of Ring • Behavior of children during the dental procedure: specifically comparing the behavior during the clinical examination and restorative procedure, object of the master dissertation of Mônica Alves Moterane, to be completed in February / 2019. • Memory of the procedure: the data have already been collected and are under analysis for a later scientific report, which will be part of the PhD thesis of Karolline Alves Viana, possibly to be completed by the end of 2019. • Salivary cortisol level: a the saliva of the participants was collected, but it is still necessary to analyze the saliva of the children and to associate it with other variables. • Analyze the child's perception about dental treatment under sedation: the data have already been collected and are under analysis for a later scientific report, which will be part of the doctoral thesis of Karolline Alves Viana, to be concluded possibly at the end of 2019 • Perform cost-effectiveness analysis of different sedative regimens. It is under development, under the responsibility of Liliani Aires Cândido Vieira. We attach, in this request, a partial report of the activities carried out so far.
Sincerely, Prof. Luciane R. R. S. Costa
